# Sugar-induced *de novo* cytokinin biosynthesis contributes to Arabidopsis growth under elevated CO_2_

**DOI:** 10.1038/s41598-019-44185-4

**Published:** 2019-05-23

**Authors:** Takatoshi Kiba, Yumiko Takebayashi, Mikiko Kojima, Hitoshi Sakakibara

**Affiliations:** 1 Department of Applied Biosciences, Graduate School of Bioagricultural Sciences, Nagoya University, Nagoya, 464-8601 Japan; 2 RIKEN Center for Sustainable Resource Science, 1-7-22, Suehiro, Tsurumi, Yokohama, 230-0045 Japan

**Keywords:** Cytokinin, Plant physiology

## Abstract

Carbon availability is a major regulatory factor in plant growth and development. Cytokinins, plant hormones that play important roles in various aspects of growth and development, have been implicated in the carbon-dependent regulation of plant growth; however, the details of their involvement remain to be elucidated. Here, we report that sugar-induced cytokinin biosynthesis plays a role in growth enhancement under elevated CO_2_ in *Arabidopsis thaliana*. Growing Arabidopsis seedlings under elevated CO_2_ resulted in an accumulation of cytokinin precursors that preceded growth enhancement. In roots, elevated CO_2_ induced two genes involved in *de novo* cytokinin biosynthesis: an adenosine phosphate-isopentenyltransferase gene, *AtIPT3*, and a cytochrome P450 monooxygenase gene, *CYP735A2*. The expression of these genes was inhibited by a photosynthesis inhibitor, DCMU, under elevated CO_2_, and was enhanced by sugar supplements, indicating that photosynthetically generated sugars are responsible for the induction. Consistently, cytokinin precursor accumulation was enhanced by sugar supplements. Cytokinin biosynthetic mutants were impaired in growth enhancement under elevated CO_2_, demonstrating the involvement of *de novo* cytokinin biosynthesis for a robust growth response. We propose that plants employ a system to regulate growth in response to elevated CO_2_ in which photosynthetically generated sugars induce *de novo* cytokinin biosynthesis for growth regulation.

## Introduction

Being sessile, plants integrate environmental and internal cues and regulate physiological and morphological processes accordingly to optimize growth and development. Because multicellular higher plants consist of organs with different functions, for example photosynthesizing leaves and roots that absorb water and inorganic nutrients, the responses must be coordinated at the whole plant level. Local as well as long-distance signalling between cells and organs via signalling molecules such as sugars and plant hormones are vital for this coordination^1–6^

Cytokinins (CKs) are a class of plant hormones that play a central role in the regulation of numerous aspects of plant growth and development acting as local and long-distance signals^7–11^. Naturally occurring CKs are mostly *N*^6^-prenylated adenine derivatives; *N*^6^-(∆^2^-isopentenyl)adenine (iP), *trans*-zeatin (tZ) and their conjugates (iP-type and tZ-type CKs, respectively) are the major forms in *Arabidopsis thaliana*^10–12^. CK activity is controlled at diverse levels, including CK quantity and modification. CK quantity is regulated mostly at the levels of *de novo* biosynthesis and degradation catalysed by adenosine phosphate-isopentenyltransferase (IPT) and CK oxidase/dehydrogenase (CKX), respectively^13–16^. Side-chain modification to form tZ-type CKs by cytochrome P450 monooxygenase CYP735A specifies CK activity toward shoot growth^17,18^. Recently, CK translocation via the vascular system was reported to also be important^19–21^. Shoot-to-root translocation of CK via phloem is critical for root vascular patterning, whereas root-to-shoot translocation via xylem mediated by ABCG14 regulates shoot growth and development. Regulation of CK activity is relevant to various plant developmental processes and environmental responses such as shoot apical meristem activity, branching, stress and nutritional responses^22–28^.

Because plants are autotrophs that rely on photosynthesis to gain most of their building materials and energy, carbon availability is a major factor defining plant growth and development^29–32^. To maximize fitness, long-distance communication is required for plants to balance the growth of photosynthesizing leaves and that of carbon consuming roots in response to carbon availability^6,33^. In various plant species, elevated CO_2_ (i.e. high carbon availability) generally results in growth acceleration of both shoots and roots, although the root-to-shoot mass ratios are variable depending on species and environmental conditions^25,26,29,34–37^. CKs have been implicated in growth acceleration because cell division and cell differentiation in the meristem are influenced by CKs and are often accompanied by CK accumulation^26,38^. In addition, an increase in tZ-type CKs was detected in the xylem sap of cotton and tobacco plants grown under elevated CO_2_, implying that tZ-type CKs have a role as root-to-shoot signals under elevated CO_2_ conditions^25,39^. However, how CKs accumulate and whether the accumulation and root-to-shoot translocation of CK is relevant to growth acceleration under elevated CO_2_ (i.e. high carbon availability) remains to be determined.

In this study, we revealed that enhancement of *de novo* biosynthesis is responsible for CK accumulation under elevated CO_2_ and that the enhancement is triggered by sugars derived from photosynthesis. Detailed growth analyses of mutants defective in cytokinin *de novo* biosynthesis (*ipt3 ipt5 ipt7* and *cyp735a1 cyp735a2*) revealed that accumulation of tZ-type cytokinins through *de novo* biosynthesis plays a role in a robust growth response to elevated CO_2_ by both shoots and roots. Altogether, these results suggest that the *de novo* tZ-type CK biosynthesis triggered by photosynthetically generated sugars contributes to growth enhancement under elevated CO_2_ in *Arabidopsis*.

## Results

### Elevated CO_2_ increases cytokinin precursor concentrations in shoots and roots

**Figure 1:**
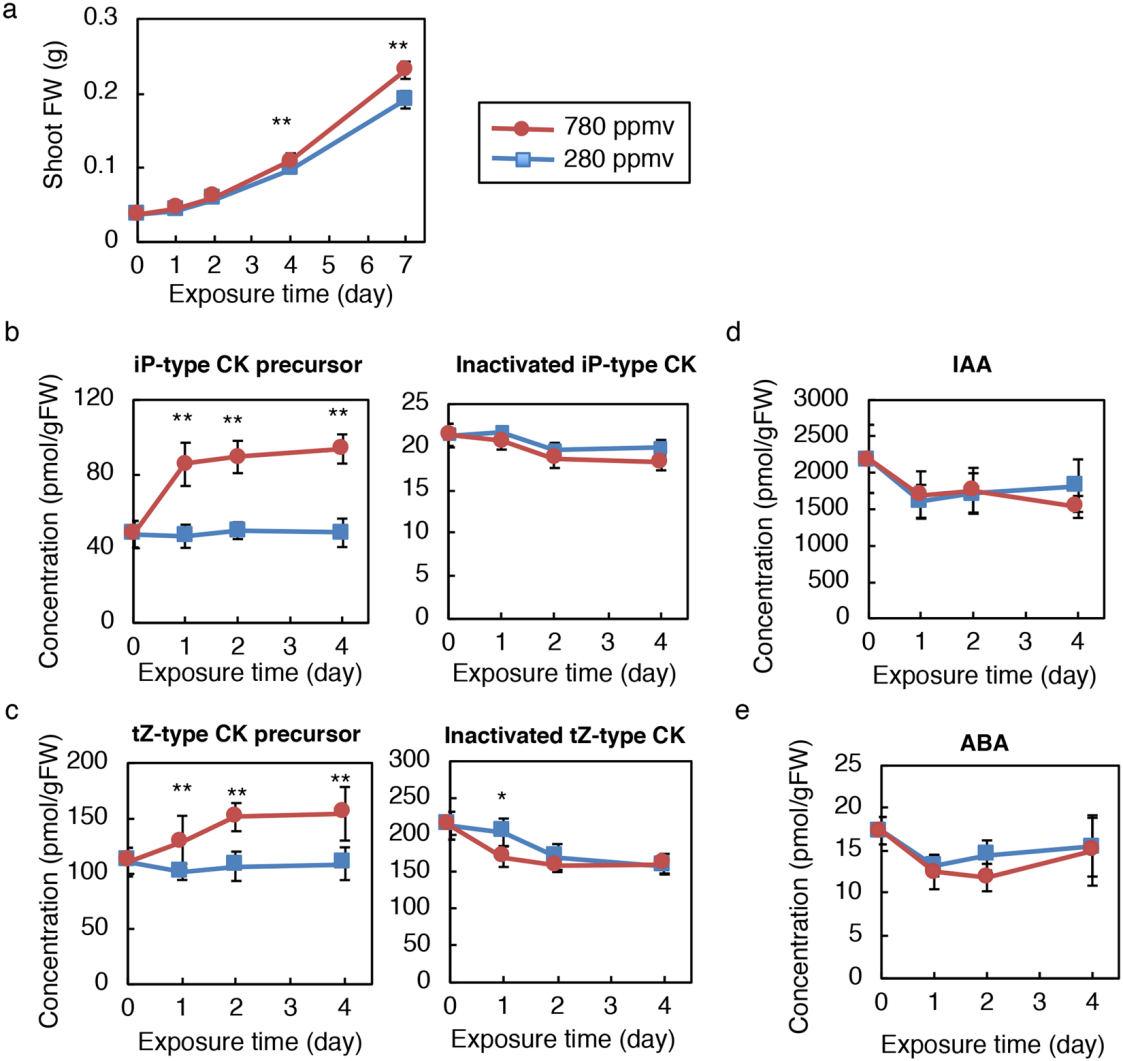
Effects of high CO_2_ on growth and hormone concentrations in soil-grown plants. Shoot fresh weight (**a**), concentrations of iP-type cytokinin (CK) precursors and inactivated iP-type CKs (**b**), concentrations of tZ-type CK precursors and inactivated tZ-type CKs (**c**), IAA concentration (**d**), and ABA concentration (**e**) of Col-0 shoots incubated at 280 ppmv or 780 ppmv CO_2_ for the indicated periods. Error bars represent standard deviations (**a**, n = 10; **b**–**e**, n = 8). Asterisks indicate statistically significant differences between 280 ppmv CO_2_- and 780 ppmv CO_2_-treated samples at the same exposure time (*p < 0.05; **p < 0.01; Student’s *t*-test). FW, fresh weight; tZ, *trans*-zeatin; iP, *N*^*6*^-(*∆*2-isopentenyl)adenine; iP-type CK precursor, sum of iPR and iPRPs; inactivated iP-type CK, sum of iP7G and iP9G; tZ-type CK precursor, sum of tZR and tZRPs; inactivated tZ-type CK, sum of tZ7G, tZZ9G, tZOG, tZROG, and tZRPsOG. The concentrations of all quantified hormones are shown in Supplementary Table S1.

To examine the effects of elevated CO_2_ on growth and CK levels, plants were grown under low [280 parts per million by volume (ppmv)] and high CO_2_ (780 ppmv) on soil. Two hundred and eighty ppmv is the pre-industrial atmospheric concentration and 780 ppmv is a value close to the median of values predicted at the end of this century^40^. When wild-type *Arabidopsis* Col-0 were germinated and grown under low or high CO_2_ with a 12 h light/12 h dark photoperiod for four weeks, high CO_2_-grown plants deposited more biomass and developed more leaf area and rosette leaves than low CO_2_-grown plants did, as described previously (Supplementary Fig. S1^32,41,42^). Using the same growth conditions, we analysed changes in the CK concentration following exposure to high CO_2_. Sixteen-day-old Col-0 plants grown in low CO_2_ were transferred to low or high CO_2_, and CK concentrations in the whole shoot were followed for four days. Under these conditions, significant differences in shoot fresh weight between high and low CO_2_-treated plants became evident from day 4 onward (Fig. 1a). The levels of iP-type CK precursors (iPR and iPRPs) and tZ-type CK precursors (tZR and tZRPs) in high CO_2_-treated shoots increased after one day and stayed high until day 4 compared with those of low CO_2_-treated plants (Fig. 1b,c). On the other hand, concentrations of other CK metabolites including inactivated iP-type CKs (iP7G and iP9G), and tZ-type CKs (tZ7G, tZ9G, tZOG, tZROG, and tZRPsOG) did not change consistently during the period of observation (Fig. 1b,c; Supplementary Table S1). Furthermore, the high CO_2_-treatment did not significantly affect the levels of other plant hormones, including a gibberellin precursor (GA_24_), IAA, and ABA (Fig. 1d,e; Supplementary Table S1). These results showed that iP-type and tZ-type CK precursors accumulate in the shoot prior to growth enhancement at high CO_2_ under our experimental conditions.

**Figure 2:**
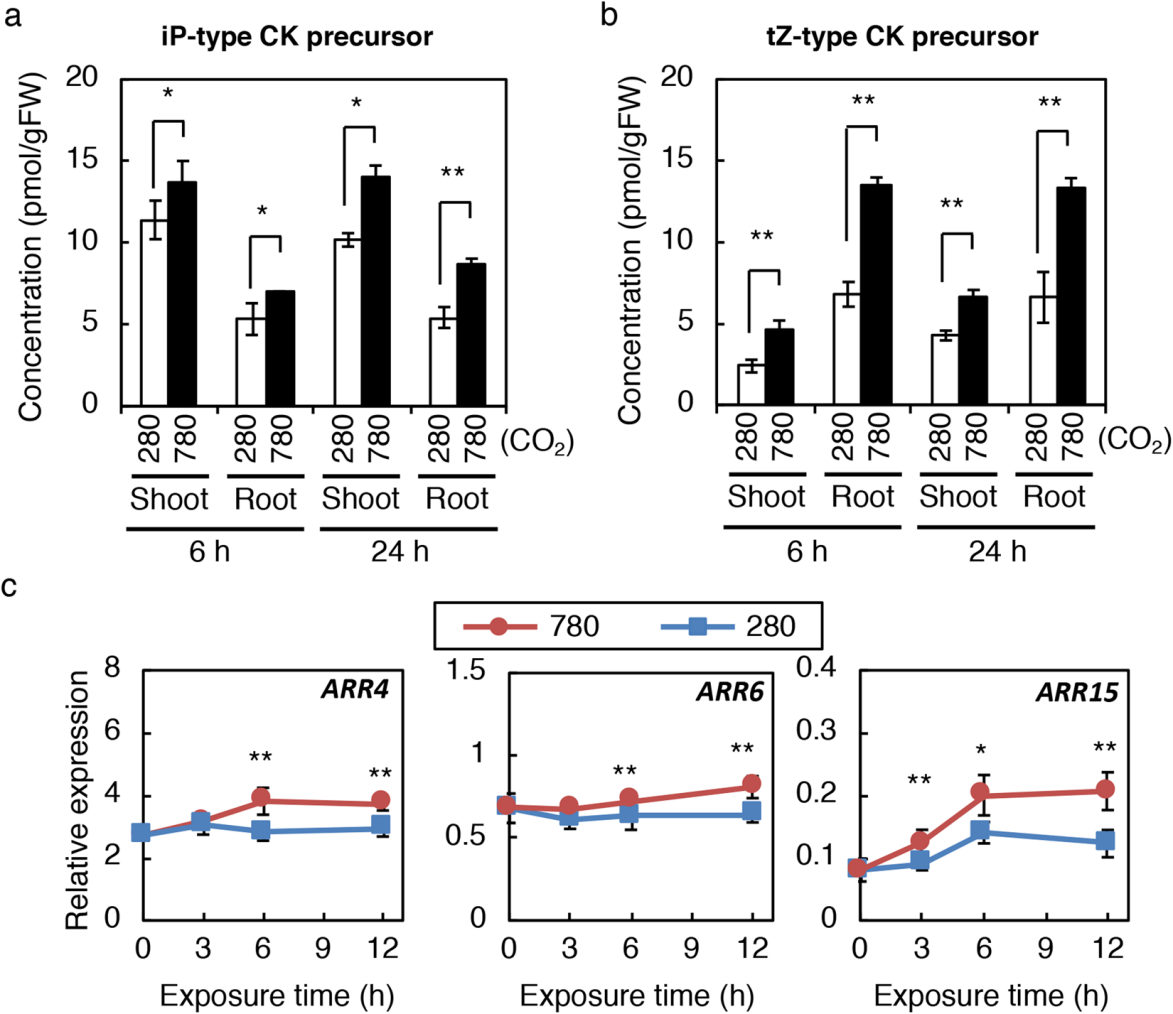
Cytokinin levels and activity in seedlings exposed to high CO_2_. (**a**,**b**) Cytokinin (CK) levels in shoots and roots of Col-0 seedlings exposed to low and high CO_2_. iP-type CK precursor levels (**a**) and tZ-type CK precursor levels (**b**) in shoots and roots are presented. (**c**) Expression levels of type-A *ARR* genes in Col-0 seedlings exposed to low and high CO_2_. Transcript levels of *ARR4*, *ARR6*, and *ARR15* were analysed by quantitative RT-PCR. Expression levels were normalized using *At4g34270* as an internal control. Twelve-day-old seedlings grown on 1/2 MS agar plates at 280 ppmv were exposed to 280 ppmv (280) or 780 ppmv (780) CO_2_ for the indicated periods. Error bars represent standard deviations of three biological replicates. Asterisks indicate statistically significant differences between 280 ppmv CO_2_- and 780 ppmv CO_2_-treated samples at the same exposure time (*p < 0.05; **p < 0.01; Student’s *t*-test). FW, fresh weight; tZ, *trans*-zeatin; iP, *N*^*6*^-(*∆*^2^-isopentenyl)adenine. The concentrations of cytokinin molecular species are shown in Supplementary Table S2.

Next, we employed a growth system in which Col-0 seedlings were germinated and grown on half-strength MS (1/2 MS) agar plates placed vertically to allow the analysis of both shoots and roots. Twelve-day-old wild-type seedlings grown under continuous light in low CO_2_ were transferred to low or high CO_2_, and the CK concentrations in shoots and roots were measured after 6 h and 24 h. The basal level of tZ, tZRPs and tZ-N-conjugates in this measurement (Supplementary Table S2) was very different from that in soil-grown plants (Supplementary Table S1). This is possibly due to differences in growth conditions and plant ages, as a similar trend has been observed previously^17^. Accumulation of tZ, and iP-type and tZ-type precursors became evident in shoots and roots as early as 6 h after commencing the high CO_2_-treatment and continued until 24 h, whereas the levels of other CK metabolites did not consistently change (Fig. 2a,b; Supplementary Table S2).

It is known that the accumulation of CK precursors generally results in increased CK activity^7,43^. To verify that CK signalling is activated in parallel with precursor accumulation, the expression of immediate-early CK responsive type-A *ARR* genes was analysed in whole seedlings treated as in Fig. 2a. As expected, *ARR4*, *ARR*6, and *ARR15* were induced, with the timing of induction similar to that of CK precursor accumulation (Fig. 2c). Since recent studies on plant membrane binding and crystal structure analysis showed that precursors do not bind to Arabidopsis CK receptors^44,45^, one would expect that active CKs (iP and tZ) are accumulated in response to an increase in CK precursor levels. However, active CKs were not always increased significantly in our experiments (for example, Supplementary Tables S1, S2). This lack of significant change in active CK levels has been reported previously^46,47^ and we assume that it is because only a fraction of active CKs exists in a compartment where they can be perceived by CK receptors. Taken together, these results indicated that elevated CO_2_ resulted in increased CK activity, which is triggered by CK precursor accumulation in shoots and roots.

### Cytokinin biosynthetic genes, *AtIPT3* and *CYP735A2*, are up-regulated in roots under elevated CO_2_

**Figure 3:**
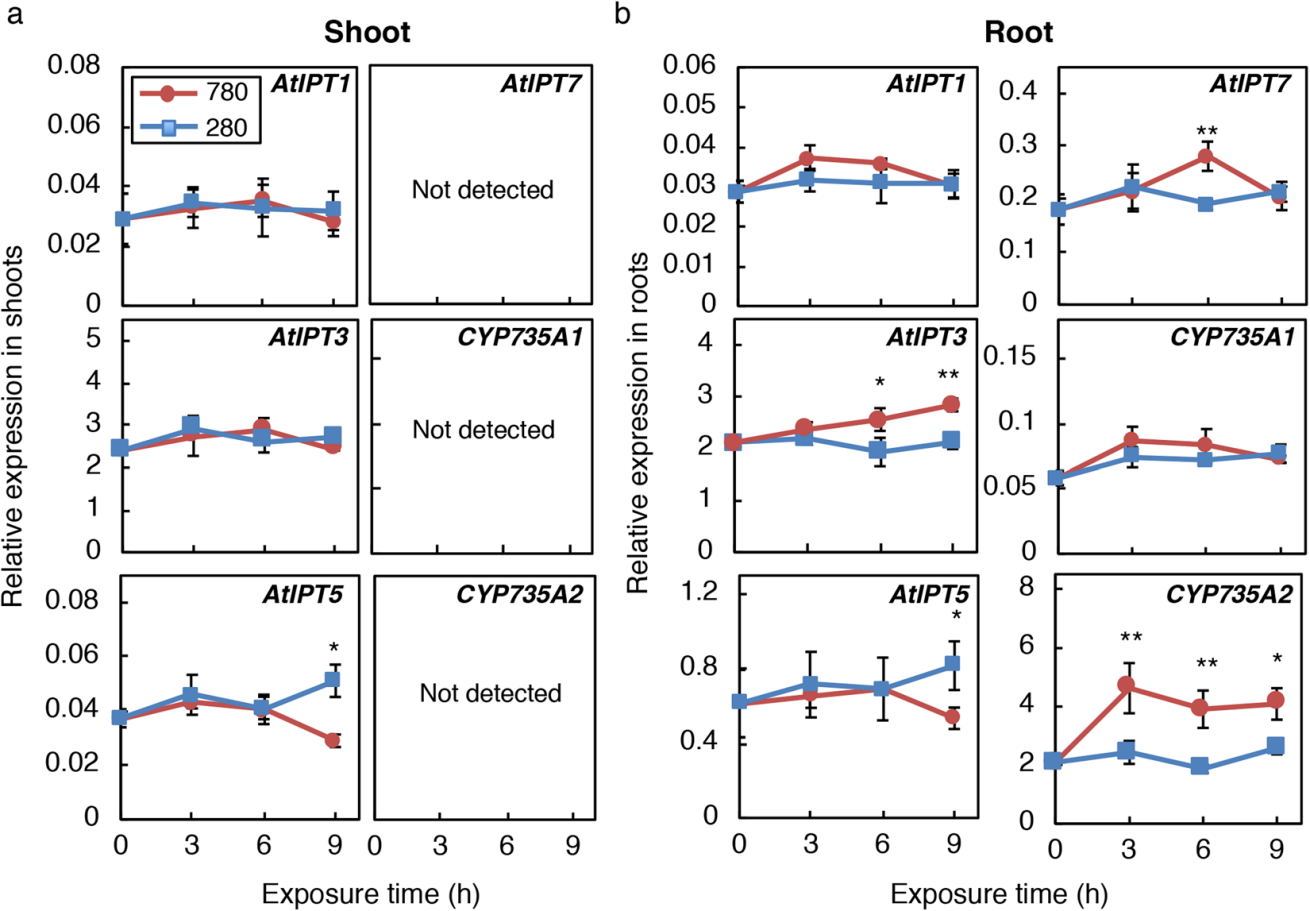
Expression of genes involved in cytokinin biosynthesis in shoots and roots upon exposure to high CO_2_. Transcript levels of *AtIPT1*, *AtIPT3*, *AtIPT4*, *AtIPT5*, *AtIPT6*, *AtIPT7*, *AtIPT8*, *CYP735A1*, and *CYP735A2* were analysed in shoots (**a**) and roots (**b**) of Col-0 seedlings by quantitative RT-PCR. Expression levels of *AtIPT4*, *AtIPT6*, *and AtIPT8* were below the detection limit in shoots and roots. Expression levels were normalized using *At4g34270* as an internal control. Twelve-day-old seedlings grown on 1/2 MS agar plates at 280 ppmv were exposed to 280 ppmv (280) or 780 ppmv (780) CO_2_ for the indicated periods. Error bars represent standard deviations of three biological replicates. Asterisks indicate statistically significant differences between 280 ppmv CO_2_- and 780 ppmv CO_2_-treated samples at the same exposure time (**p < 0.01; *p < 0.05; Student’s *t*-test).

Generally the accumulation of CK precursors reflects increased *de novo* biosynthesis^7,43^. The initial step of *de novo* CK biosynthesis is catalysed by IPT^13,14^, and the key step of tZ-type *de novo* CK biosynthesis requires CYP735A^17,18^. We examined the expression levels of seven *IPT* (*AtIPT1*, *AtIPT3*, *AtIPT4*, *AtIPT5*, *AtIPT*6, *AtIPT7*, *AtIPT8*) and two *CYP735A* (*CYP735A1* and *CYP735A2*) genes in *Arabidopsis* shoots and roots of Col-0 seedlings incubated at low or high CO_2_ from 3 h, an earlier time point than when CK precursor accumulation was observed (up to 9 h). *AtIPT4*, *AtIPT*6, and *AtIPT8* were not detected in shoots nor roots in our experimental conditions. In shoots, none of the genes examined were affected by high CO_2_ except for *AtIPT5* that was down-regulated at 9 h (Fig. 3a). In roots, the transcript level of *CYP735A2* increased after 3 h and stayed high till 9 h and that of *AtIPT3* steadily accumulated after the onset of high CO_2_ treatment (Fig. 3b). On the other hand, the levels of the other transcripts remained unchanged or showed transient fluctuations (Fig. 3b). Down-regulation of *AtIPT5* in both shoot and root might be caused by accumulated CK because *AtIPT5* has been reported to be repressed by CK^48^. Since CK levels are determined by the balance between *de novo* biosynthesis and degradation, we also analysed the expression of genes encoding CK-degrading enzymes, *CKX*. Among seven *CKX*s in *Arabidopsis*, the expression of six genes was detected but none of these genes were down-regulated in shoots and roots under high CO_2_ treatment (Supplementary Fig. S2). Rather, the expression of *CKX1*, *CKX4*, *CKX6*, and *CKX7* was transiently enhanced, possibly in response to CK accumulation (Supplementary Fig. S2). Similar CK precursor accumulation and induction of *AtIPT3* and *CYP735A2* were observed when seedlings were grown and treated under 12-h-light/12-h-dark cycles (Supplementary Fig. S3; Supplementary Table S3). These results suggested that the induction of *AtIPT3* and *CYP735A2* in roots plays a role in iP- and tZ-type CK precursor accumulation under elevated CO_2_.

### Photosynthetically generated sugars induce *AtIPT3* and *CYP735A2* in roots

**Figure 4:**
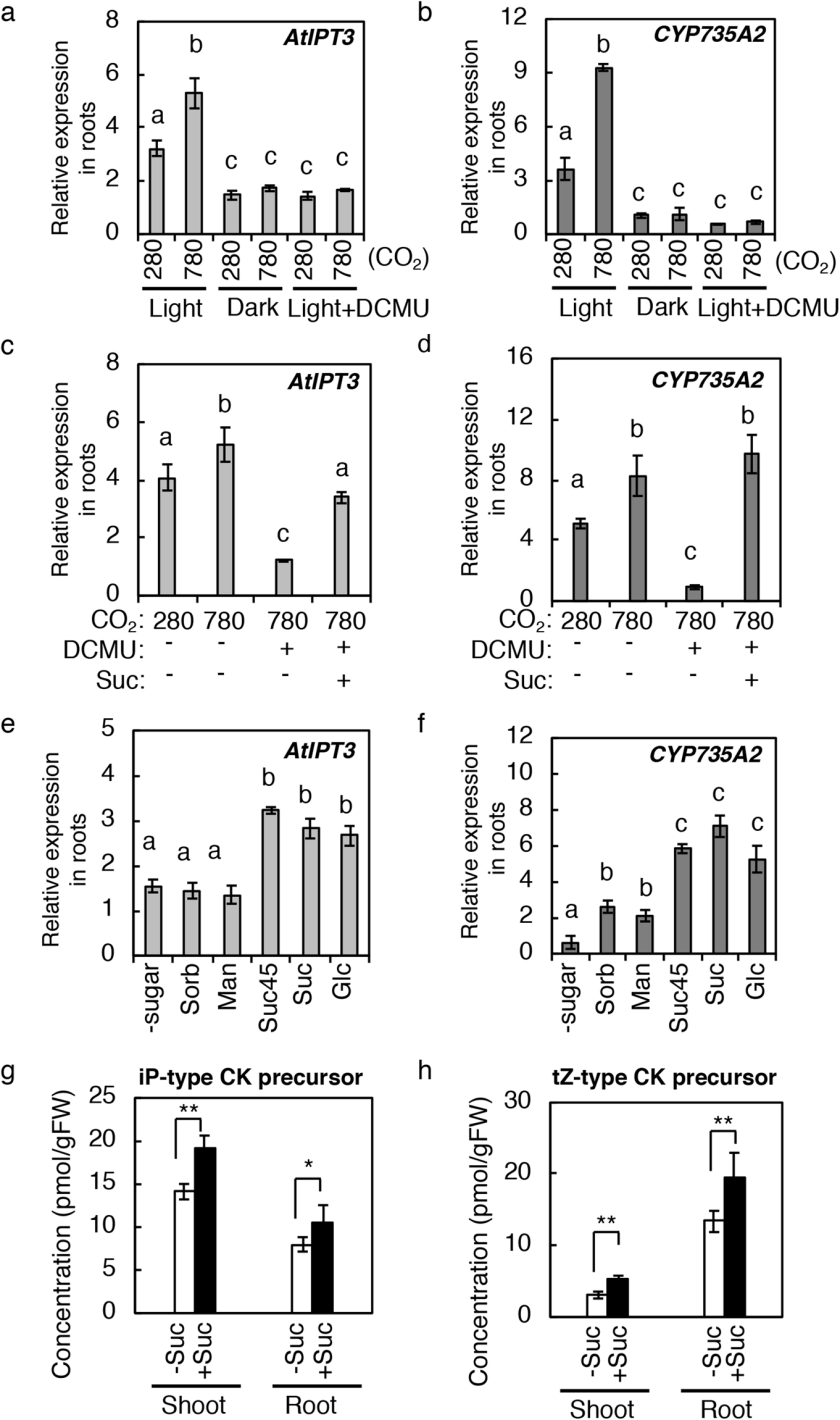
Effects of photosynthesis and sugars on the expression of *AtIPT3* and *CYP735A2*, and cytokinin levels. (**a**,**b**) Effects of dark and DCMU on *AtIPT3* (a) and *CYP735A2* (b) expression in Col-0 roots. Seedlings were exposed to 280 ppmv or 780 ppmv CO_2_ under light (Light), under light with 40 µM DCMU (Light + DCMU), or in the dark (Dark). (**c**,**d**) *AtIPT3* (c) and *CYP735A2* (d) expression in Col-0 seedlings treated with 40 µM DCMU in the presence (+) or absence (−) of 90 mM sucrose (Suc) and/or DCMU for six hours. (**e**,**f**) Effects of sugars on the expression of *AtIPT3* (e) and *CYP735A2* (f) in Col-0 roots. Seedlings were incubated on plates with 90 mM sorbitol (Sorb), mannitol (Man), sucrose (Suc), glucose (Glc), with 45 mM sucrose (Suc45), or without sugar (-sugar) for six hours at 280 ppmv CO_2_ in the dark. (**g**,**h**) Changes in cytokinin levels in seedlings treated with sucrose. iP-type CK precursor levels (g) and tZ-type CK precursor levels (h) in shoots and roots are presented. Twelve-day-old seedlings grown on 1/2 MS agar plates at 280 ppmv were treated with 45 mM sucrose (+Suc) or without sucrose (−Suc) at 280 ppmv for 24 h. The concentrations of cytokinin molecular species are shown in Supplementary Table S3. Asterisks indicate statistically significant differences (*p < 0.05; Student’s *t*-test). Error bars represent standard deviations of four biological replicates. Asterisks indicate statistically significant differences (*p < 0.05; Student’s *t*-test). Different lower-case letters indicate statistically significant differences as indicated by Tukey’s HSD test (*p* < 0.05). Expression levels were analysed by quantitative RT-PCR and normalized using *At4g34270* as an internal control.

Next, we tested the involvement of photosynthesis in the induction of *AtIPT3* and *CYP735A2* by incubating wild-type seedlings in the dark or by applying the photosynthesis inhibitor DCMU, which blocks electron flow from photosystem II. When seedlings were incubated in the dark or in the light with DCMU at 280 ppmv for 6 h, expression levels of *AtIPT3* and *CYP735A2* were reduced compared to the control (Fig. 4a,b). The induction of *AtIPT3* and *CYP735A2* in response to elevated CO_2_ was completely abolished by these treatments (Fig. 4a,b), indicating that photosynthetic activity is required for the maintenance and induction of *AtIPT3* and *CYP735A2* expression.

Elevated CO_2_-treatment reportedly increases endogenous sugar concentrations (e.g. fructose, glucose, and sucrose), whereas DCMU treatment reduces sugar levels^31,37,49^. To examine whether the DCMU-triggered attenuation of *AtIPT3* and *CYP735A2* induction were caused by lowered levels of sugars, we supplemented DCMU-treated seedlings with sucrose. Sucrose reversed the effect of DCMU on *AtIPT3* and *CYP735A2* expression (Fig. 4c,d). We also tested the effects of other sugars on *AtIPT3* and *CYP735A* expression. Seedlings were transferred to agar plates containing metabolizable sugars (sucrose and glucose) or non-metabolizable sugars (sorbitol and mannitol) and were incubated at 280 ppmv CO_2_ in the dark. Metabolizable sugars were able to induce *AtIPT3* and *CYP735A* expression (Fig. 4e,f). On the other hand, non-metabolizable sugars were ineffective in inducing *AtIPT3* expression (Fig. 4e). *CYP735A2* expression was induced by non-metabolizable sugars but to a much lower extent compared with metabolizable sugars (Fig. 4f). Since *CYP735A2* seems to be moderately activated by osmotic stress^50^, the induction by non-metabolizable sugars is probably due to osmotic effects.

**Figure 5:**
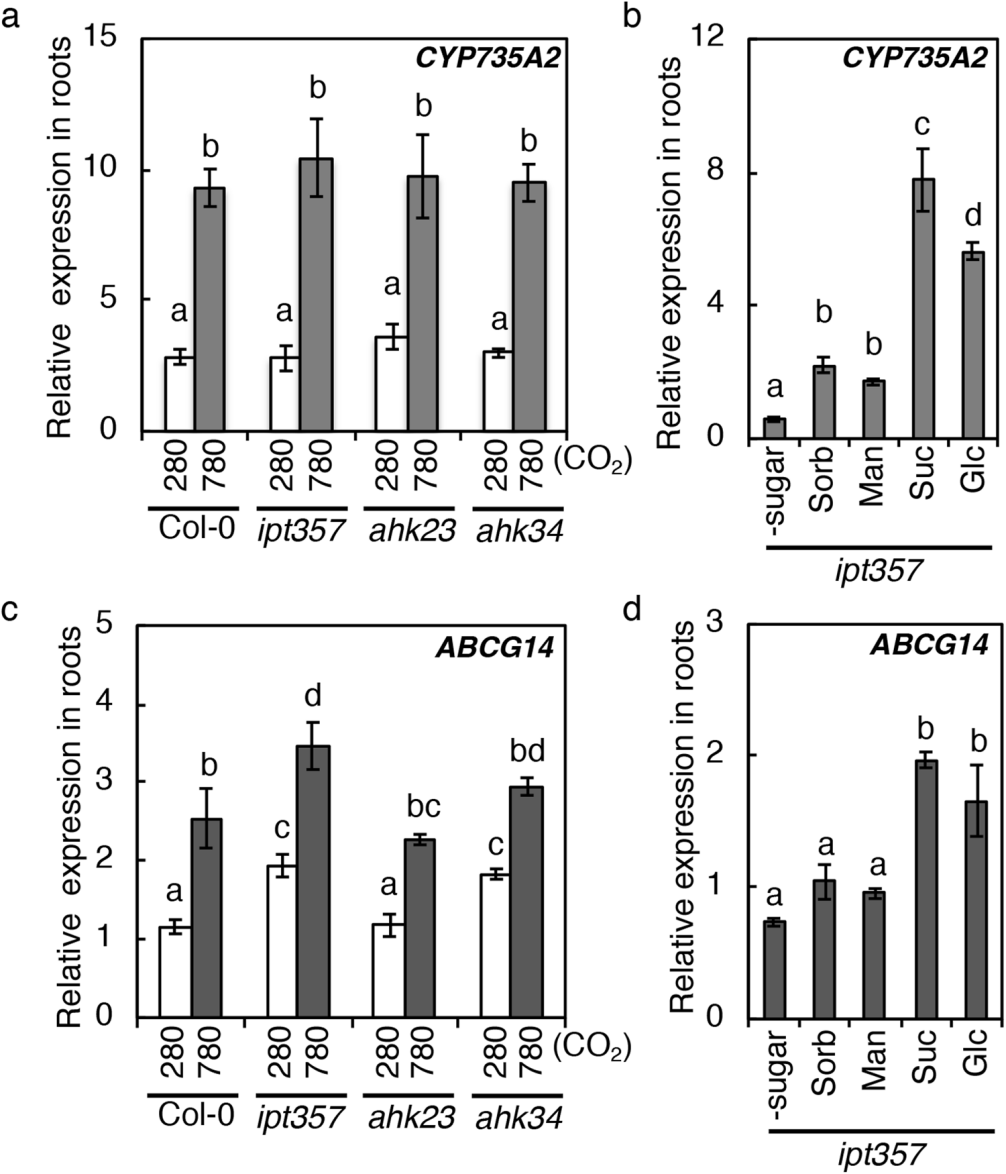
Expression of *CYP735A2* and *ABCG14* in cytokinin biosynthetic and signaling mutants treated with high CO_2_ or sugars. (**a**,**c**) Wild-type (Col-0), *ipt3 ipt5 ipt7* (*ipt357*), *ahk2 ahk3* (*ahk23*) and *ahk3 ahk4* (*ahk34*) seedlings grown on 1/2MS agar plates at 280 ppmv CO_2_ for 12 days were exposed to 280 ppmv or 780 ppmv for six hours and then roots were harvested. (**b**,**d**) *ipt357* seedlings were transferred to new plates containing 90 mM of sorbitol (Sorb), mannitol (Man), sucrose (Suc) or glucose (Glc), or without any sugar (-sugar). Roots were harvested after six hours. Expression levels were analysed by quantitative real-time PCR and normalized using *At4g34270* as an internal control. Error bars represent standard deviation of three biological replicates. Different lower-case letters indicate statistically significant differences as indicated by Tukey’s HSD test (*p* < 0.01).

*CYP735A2* is known to be a CK-inducible gene^18,21^. Thus we tested whether *CYP735A2* induction by elevated CO_2_ and sugars is the result of accumulated CKs by employing *ipt3 ipt5 ipt7* (*ipt357)* and the cytokinin receptor mutants *ahk2 ahk3* and *ahk3 ahk4*^51–53^ that are defective in CK biosynthesis and signalling, respectively. Elevated CO_2_ and sugars induced *CYP735A2* expression in the mutants at a level comparable to Col-0 (Fig. 5a,b), indicating that sugars induce this gene independently of CK.

These results suggested that *AtIPT3* and *CYP735A2* are induced in roots under elevated CO_2_ by sugars generated in shoots by photosynthesis. Consistent with this, sucrose treatment resulted in an accumulation of CK precursors in shoots and roots (Fig. 4g,h; Supplementary Table S4).

### Photosynthetically generated sugars induce cytokinin precursor accumulation irrespective of the nitrate status

**Figure 6:**
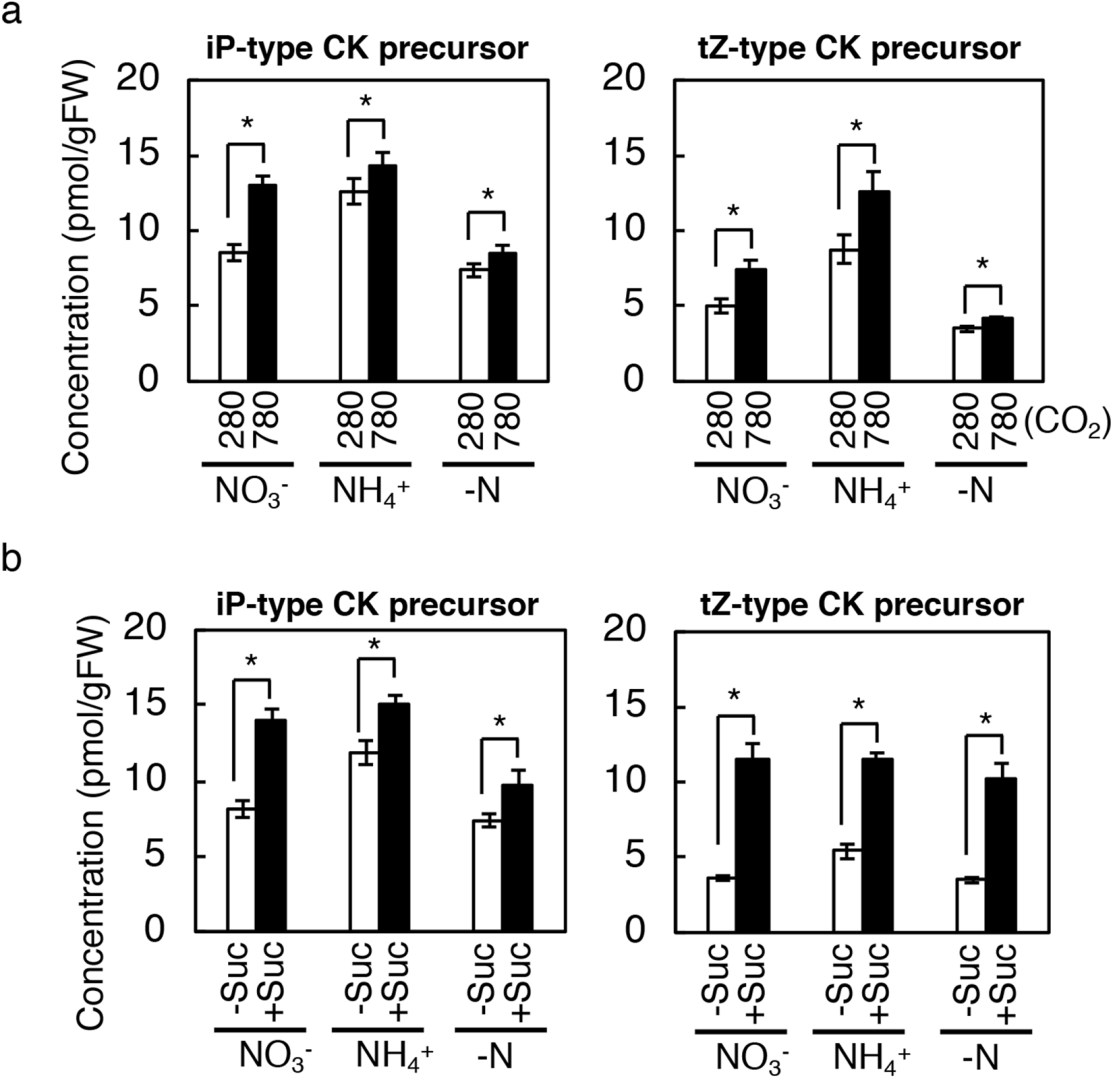
Cytokinin levels in wild-type seedlings exposed to high CO_2_ or treated with sucrose under different nitrogen nutrient conditions. (**a**) Cytokinin (CK) levels in wild-type (Col-0) whole seedlings exposed to low or high CO_2_ under different nitrogen nutrient conditions. Seedlings were exposed to 280 ppmv (280) or 780 ppmv (780) CO_2_ for 24 h on modified 1/2 MS agar plates containing 10 mM KNO_3_ (NO_3_^−^) or 10 mM NH_4_Cl (NH_4_^+^), or without any nitrogen source (-N). The concentrations of cytokinin molecular species are shown in Supplementary Table S5. (**b**) Cytokinin levels in wild-type (Col-0) whole seedlings treated with (+Suc) or without (−Suc) 45 mM sucrose for 24 h on modified 1/2 MS agar plates containing 10 mM KNO_3_ (NO_3_^−^) or 10 mM NH_4_Cl (NH_4_^+^), or without any nitrogen source (-N). The concentrations of cytokinin molecular species are shown in Supplementary Table S6. Twelve-day-old seedlings grown at 280 ppmv were used. Error bars represent standard deviations of four biological replicates. Asterisks indicate statistically significant differences (*p < 0.05; Student’s *t*-test). FW, fresh weight; tZ, *trans*-zeatin; iP, *N*^*6*^-(*∆*^2^-isopentenyl)adenine.

It is known that *de novo* CK biosynthesis is regulated by nitrate in *Arabidopsis*^54^. Since carbon availability is reported to influence nitrate transporter gene expression and nitrate uptake^55,56^, it is possible that carbon availability affects CK biosynthesis indirectly through nitrate-related pathways. To test this possibility, we measured CK levels in wild-type seedlings treated with high CO_2_ or sucrose, and with and without nitrate. Twelve-day-old seedlings were treated with high CO_2_ or sucrose on agar plates containing 10 mM KNO_3_, 10 mM NH_4_Cl, or no nitrogen source, and the CK concentrations in the whole seedling were measured after 24 h. The levels of CK precursors increased in all nitrogen conditions tested in response to high CO_2_ or sucrose treatment (Fig. 6; Supplementary Tables S5 and S6), suggesting that sugars induce CK precursor accumulation independent of nitrate-related pathways.

### The *ipt3 cyp735a2* mutant still accumulates cytokinin precursors in response to elevated CO_2_

Having shown the relevance of *AtIPT3* and *CYP735A2* in the elevated CO_2_-enhanced *de novo* CK biosynthesis, we investigated whether these processes contribute to growth enhancement under elevated CO_2_ by generating an *ipt3 cyp735a2* double mutant. To this end, 12-day-old seedlings grown on agar plates were incubated at low or high CO_2_ for seven days. Fresh weight (FW) was measured before and after the treatment, and relative growth rate (RGR) was calculated. Growth differences of Col-0 between low CO_2_- and high CO_2_-incubated seedlings were clearly observed; the FW and RGR of both the shoot and the root were significantly increased by the high CO_2_ treatment (Supplementary Fig. S4a–c). However, no significant difference in the FW and RGR was observed between the double mutant and WT (Supplementary Fig. S4a–c). To understand this lack of growth phenotype, we analysed changes in iP- and tZ-type precursor CK levels in shoots and roots of the double mutant following exposure to high CO_2_. Under low CO_2_, the double mutant contained significantly reduced levels of iP-type CK precursors in shoots (Supplementary Fig. S4d; Supplementary Table S7). However, it accumulated both CK precursors in both organs in response to high CO_2_-treatment, though the levels of accumulation were generally lower compared with WT (Supplementary Fig. S4d,e; Supplementary Table S7), indicating that *AtIPT3* and *CYP735A2* are not the only factors mediating the elevated CO_2_-induced CK precursor accumulation. Together, these results suggest that the double mutant lacks a growth phenotype because it still is able to accumulate enough CKs for elevated CO_2_-triggered growth enhancement.

### The *ipt3 ipt5 ipt7* and *cyp735a1 cyp735a2* mutants are impaired in elevated CO_2_-triggered growth enhancement

**Figure 7:**
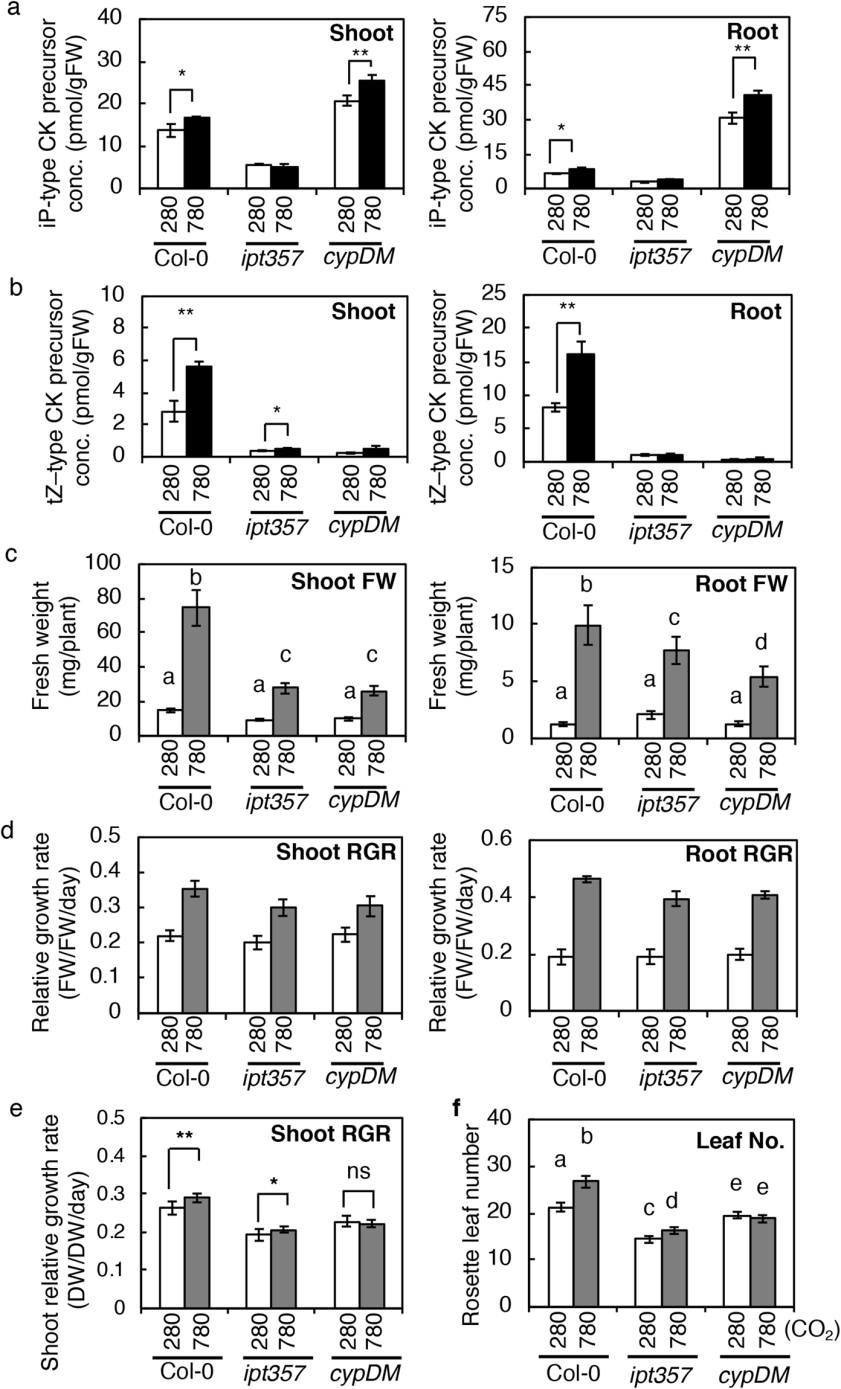
Cytokinin levels and growth of wild-type, *ipt3 ipt5 ipt7* and *cyp735a1 cyp735a2* seedlings exposed to high CO_2_. (**a**,**b**) The concentration of iP-type cytokinin (CK) precursors (a) and tZ-type CK precursors (b) in shoots and roots of wild-type (Col-0), *ipt3 ipt5 ipt7* (*ipt357*) and *cyp735a1 cyp735a2* (*cypDM*) plants exposed to 280 ppmv (280) or 780 ppmv (780) CO_2_ for 24 h. Asterisks indicate statistically significant differences (**p < 0.01; *p < 0.05; Student’s *t*-test). The concentrations of cytokinin molecular species are shown in Supplementary Table S7. (**c**,**d**) Fresh-weight (c) and relative growth rate (RGR) (d) of 19-day-old wild type (Col-0), *ipt3 ipt5 ipt7* (*ipt357*), and *cyp735a1 cyp735a2* (*cypDM*) seedlings treated under 280 ppmv (280) or 780 ppmv (780) CO_2_ for seven days. (**d**) RGR was calculated using the fresh weight (FW) data obtained previously (Supplementary Fig. S5) and after (c) low or high CO_2_ treatment. (**e**,**f**) Shoot growth of soil-grown wild-type, *ipt3 ipt5 ipt7* and *cyp735a1 cyp735a2* plants under low or high CO_2_. (**e**) Relative growth rates (RGR) of shoots of Col-0, *ipt357*, and *cypDM* grown under 280 or 780 on soil. Dry weights of shoots shown in Supplementary Fig. 6b were used to calculate the RGR. Asterisks indicate statistically significant differences (**p < 0.001; *p < 0.01; not significant (ns), *p* > 0.01; two-way ANOVA). (**f**) Rosette leaf number of Col-0, *ipt357*, and *cypDM* counted at 31 DAG. Error bars represent standard deviations (**a**, n = 3; **b**, n = 3; **c**, n = 9; **f**, n = 10) and standard error (**d**, n = 9; **e**, n = 9). Lower-case letters denote statistically significant classes (Tukey’s HSD test, *p* < 0.05).

Since the *ipt3 cyp735a2* double mutant still accumulated CKs in response to high CO_2_ (Supplementary Fig. S4d,e), we employed higher order CK-biosynthetic mutants, *ipt357* and *cyp735a1 cyp735a2* (*cypDM*). The *ipt357* mutant lacks three major *IPT* genes^57^ and, thus, has a dramatically reduced ability to *de novo* synthesize both iP- and tZ-type CKs. The *cypDM* mutant lacks all *CYP735A* genes^17^ and, thus, is expected to accumulate iP-type CKs but not tZ-type CKs under elevated CO_2_. To verify that elevated CO_2_-induced *de novo* CK biosynthesis is attenuated in *ipt357* and *cypDM*, the CK concentrations in shoots and roots were measured. Seedlings were grown and treated as in Fig. 2 (24 h high CO_2_ treatment). In the *ipt357* mutant, the accumulation level of all CKs was relatively low compared with the wild type. The iP-type CK precursor concentrations were unaffected in shoots and roots, but the levels of tZ-type precursor CKs increased slightly in shoots with a high CO_2_ treatment (Fig. 7a,b; Supplementary Table S8). In the *cypDM* mutant, iP-type precursor CKs accumulated but tZ-type precursor CKs levels were consistently low in shoots and roots under elevated CO_2_ (Fig. 7a,b; Supplementary Table S8). These observations confirmed the inability of the mutants to accumulate CKs of the expected types under elevated CO_2_. These results showed that *de novo* CK biosynthesis, most likely mediated by *IPT3*, *IPT5*, *IPT7*, *CYP735A1* and *CYP735A2*, plays an important role in CK accumulation in response to high CO_2_.

We then investigated whether these mutants are impaired in growth enhancement under elevated CO_2_. Seedlings were grown and treated on agar plates as in Supplementary Fig. S4, and the FW was measured before and after treatment, and the RGRs were calculated. In Col-0, the FW and RGR of both the shoot and the root were dramatically increased in response to high CO_2_ (Fig. 7c,d; Supplementary Fig. S5). The *ipt357* mutant also gained more FW both in shoots and roots in high CO_2_ compared with the low CO_2_ treatment, but the extent of the increase was smaller compared with that of Col-0 (Fig. 7c). RGR analysis revealed that shoots and roots of *ipt357* grew faster in high CO_2_ than in low CO_2_ but at a lower rate compared with those of Col-0, whereas the RGR in low CO_2_ was similar among all genotypes in this growth system (Fig. 7d). Interestingly, the *cypDM* mutant displayed essentially the same growth response defects to elevated CO_2_ as the *ipt357* mutant (Fig. 7c,d), showing that accumulation of tZ-type CKs is critical for the response.

We also analysed the growth response of soil-grown plants. The mutants were germinated and grown on soil together with Col-0 under low or high CO_2_, and shoot growth was analysed at 17 and 31 days after germination (DAG) by measuring dry weight (DW). Note that it was not possible to evaluate root growth in this system. Although Col-0 plants grown in high CO_2_ had significantly higher shoot biomass compared with those grown in low CO_2_ at the beginning of analysis (17 DAG), they gained more biomass by further growth in high CO_2_ (31 DAG, Supplementary Fig. S6). RGRs between 17 and 31 DAG were significantly higher in high CO_2_-grown Col-0 plants than in the mutants (Fig. 7e). The number of rosette leaves counted on 31 DAG also significantly increased (Fig. 7f). Although the *cypDM* mutant gained more biomass under high CO_2_ (Supplementary Fig. S6), no significant change in RGR in response to high CO_2_ treatment was observed (Fig. 7e). The RGR of the *ipt357* mutant was slightly enhanced by high CO_2_ treatment (Fig. 7e). Rosette leaf numbers did not change in the *cypDM* mutant and were only marginally increased in the *ipt357* mutant (5.4 more leaves in the wild type compared with 1.9 in *ipt357*) in response to high CO_2_ (Fig. 7f). These results show that the *ipt357* and *cypDM* mutants are impaired in the acceleration of shoot growth and development under elevated CO_2_ during the growth period examined and that the *cypDM* mutant, which cannot accumulate tZ-type CKs, is severely compromised.

Together, these growth analyses suggest that CK accumulation, especially of the tZ-type, through *de novo* biosynthesis contributes to robust growth enhancement under elevated CO_2_.

### Photosynthetically generated sugars induce *ABCG14* in roots

**Figure 8:**
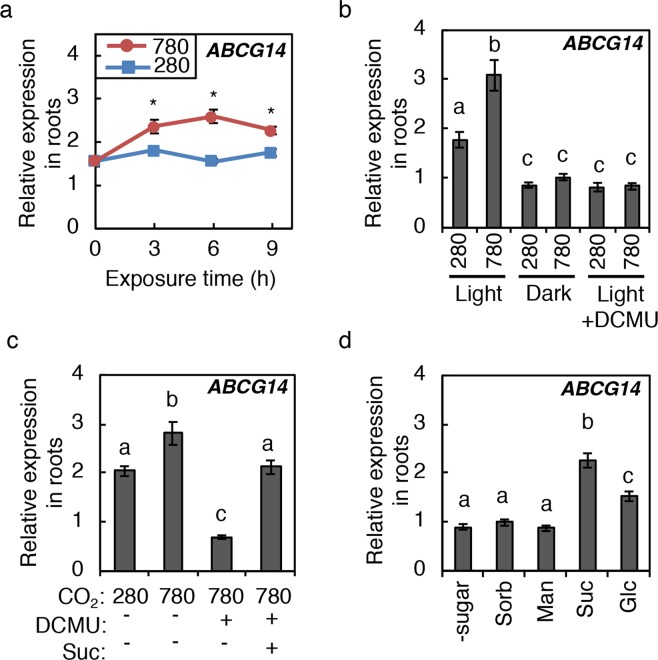
Effects of high CO_2_, photosynthesis and sugars on the expression of *ABCG14* in roots. (**a**) Expression levels of *ABCG14* in roots of Col-0 seedlings exposed to 280 ppmv (280) or 780 ppmv (780) CO_2_ for the indicated periods. Treatment was conducted as in Fig. 3. (**b**) Effects of dark and DCMU on *ABCG14* in Col-0 roots. Treatments were conducted as in Fig. 4. (**c**,**d**) Effects of sugars on *ABCG14* in Col-0 roots. Treatments were conducted as in Fig. 4. Expression levels were analysed by quantitative RT-PCR and normalized using *At4g34270* as an internal control. Error bars represent standard deviations of four biological replicates. Asterisks indicate statistically significant differences (*p < 0.05; Student’s *t*-test). Different lower-case letters indicate statistically significant differences as indicated by Tukey’s HSD test (*p* < 0.05).

It has been reported that tZ-type CKs are translocated from root to shoot by the ABCG14 protein to act as shoot growth signals^17,20,21,58^. To get insight into whether root-to-shoot translocation of CKs is relevant to the observed CK accumulation in shoots, *ABCG14* expression in roots was investigated (Fig. 8). Interestingly, *ABCG14* expression responded to high CO_2_ and sugars in a similar manner to that of *AtIPT3* and *CYP735A2* (Fig. 8). Since *ABCG14* has been reported to be CK-inducible^21^, we tested whether the CKs that accumulate in response to elevated CO_2_ and sugars are relevant to *ABCG14* induction. The *ipt357* and the cytokinin receptor mutants *ahk2 ahk3* and *ahk3 ahk4*^51–53^ were analysed as in Fig. 5a,b. *ABCG14* induction in response to elevated CO_2_ and sugars was maintained in these mutants (Fig. 5c,d), indicating that sugars induce this gene independent of CK. Together, these results suggest that root-to-shoot translocation of CKs via *ABCG14* might be involved in robust growth enhancement under elevated CO_2_ by mediating tZ-type CK accumulation in the shoot.

## Discussion

The availability of macronutrients such as nitrogen^7,43,48,59,60^, phosphate^9,61–63^ and sulphate^9,64^ affects *IPT* expression as well as CK levels. Therefore, macronutrient availability has been proposed to regulate CK levels through *de novo* biosynthesis to control plant growth and development^9,27,65^. Our investigation has revealed another pathway in which photosynthesis-derived sugars regulate *de novo* CK biosynthesis to control plant growth and development.

Our study suggests that *de novo* CK biosynthesis is triggered by photosynthetically generated sugars (Figs 1–6). There are several other reports indicating that sugars induce the expression of genes involved in the *de novo* synthesis of CKs. Transcriptome analyses show that glucose^66^ and sucrose^67,68^ treatments up-regulate *AtIPT3* and *CYP735A2*. However, how sugars are perceived (as signalling molecules, energy sources or building blocks) to induce the expression of these genes is still not understood. Thus, it is possible that sugars act indirectly through the signalling pathways of macronutrients because the metabolism of carbon and macronutrients are tightly intertwined. Although our data suggests that sugars induce CK precursor accumulation independent of nitrate-related pathways (Fig. 6), Kamada-Nobusada *et al*.^7^ reported a pathway in which the internal nitrogen status regulates CK biosynthesis. Since the internal nitrogen status can also be modulated by carbon availability, we cannot rule out the possibility that sugars affect CK biosynthesis through this pathway. Further studies on sugar and internal nitrogen sensing and signalling mechanisms are required to resolve this problem. In any case, we propose that sugars generated by photosynthesis in shoots directly or indirectly promotes *de novo* CK biosynthesis.

Under our experimental conditions, *AtIPT3* was the only gene of the *AtIPT* family induced by elevated CO_2_ and sugars (Figs 3, 4). *AtIPT3* expression is also regulated by various environmental signals to control CK levels; increases in nitrogen, phosphate, and sulphate availability induce *AtIPT3* expression^9,43,48,63^, whereas drought and salt stress repress *AtIPT3* expression^69^. Although it remains unclear – with the exception of nitrate – whether these signals regulate *AtIPT3* expression directly^70,71^, the available evidence suggests that *AtIPT3* functions to integrate and translate various signals in the root into *de novo* CK biosynthetic activity. We also found that *CYP735A2* and *ABCG14* are high CO_2_- and sugar-inducible (Figs 3, 4, 5, 8). Although *CYP735A2* and *ABCG14* are known to be CK-inducible genes^18,21^, we showed that sugars induce these genes independent of CK (Fig. 5). These results indicate that *CYP735A2* and *ABCG14* are controlled by two independent signals: shoot-derived signals (sugars) and root internal cues (root-synthesized CKs) in the response to elevated CO_2_. Thus, *CYP735A2* and *ABCG14* might act to integrate signals from shoots and roots and translate these signals into tZ-type CKs translocated from root to shoot. However, it remains to be determined whether ABCG14-mediated root-to-shoot translocation activity is regulated at the level of expression or by some other means.

In our expression analysis, *AtIPT3* and *CYP735A2* were the only genes induced under elevated CO_2_ among the *de novo* CK biosynthetic genes (Fig. 3). However, the *ipt3 cyp735a2* double mutant still accumulated CKs, although at a lower level than WT (Supplementary Fig. S4d,e). The *ipt357* and *cypDM* mutants were unable to accumulate iP-type and tZ-type CKs, respectively, in response to high CO_2,_ suggesting that not only *AtIPT3* and *CYP735A2* but also *AtIPT5*, *AtIPT7*, and *CYP735A1* are involved in the accumulation. Since these genes were not found to be regulated at the level of transcript accumulation, post-transcriptional regulation might be involved. Consistently, it has been reported that AtIPT3 farnesylation modulates this protein’s subcellular localization and enzymatic properties^72^. It should be noted that we cannot exclude that other genes involved in CK biosynthesis, modification, and/or degradation, and/or post-transcriptional regulation might be relevant to the accumulation of CKs.

In this study, the role of CKs in growth enhancement under elevated CO_2_ was evaluated by analysing the growth of *ipt357*, a mutant deficient in iP- and tZ-type CKs, and *cypDM*, a mutant deficient in tZ-type CKs. Both mutants displayed similar growth response defects (Fig. 7), indicating that tZ-type CKs are required for robust growth enhancement of shoots and roots under elevated CO_2_. A reduction in shoot growth acceleration in these mutants is consistent with previous reports that tZ-type CKs and their root-to-shoot translocation act to promote shoot growth^17,20,21^. However, a reduction in root growth acceleration cannot be explained by CK action because CKs generally act to repress root growth^73,74^. This result suggests that CK is not the major determinant of root growth rate. It is plausible that slowed root growth is a consequence of reduced photosynthesis (as sources of energy and building blocks) by smaller shoots, but it is also possible that complex crosstalk might exist between CK and sugars.

Here, we revealed that sugar-induced *de novo* biosynthesis of CKs plays a role in the robust growth enhancement under elevated CO_2_. This finding provides some insight into the mechanisms that plants employ to optimise growth in a fluctuating environment. Taking into account that *AtIPT3*, *CYP735A2*, and *ABCG14* are induced in the root by photosynthetically generated sugars (Figs 3, 4, 5, 8), it is tempting to speculate that there is a systemic growth regulatory mechanism in which photosynthetically generated sugars induce *de novo* tZ-type CK biosynthesis in the root and root-to-shoot translocation of the CK via ABCG14 for growth regulation of the shoot.

## Materials and Methods

### Plant material and growth conditions

*Arabidopsis thaliana* ecotype Columbia (Col-0) was used as the wild type. The cytokinin biosynthetic triple mutants *ipt3 ipt5 ipt7*^57^, the cytokinin receptor double mutants *ahk2 ahk3* and *ahk3 ank4*^53^, and the *cyp735a1-2 cyp735a2-2* double mutant^17^ were characterized previously. The *ipt3 cyp735a2-1* and *ipt3 cyp735a2-2* double mutants were generated by crosses between the *ipt3 ipt5 ipt7* and the *cyp735a1-2 cyp735a2-1* mutant, and *cyp735a1-2 cyp735a2-2* mutants. For studies on soil-grown plants, stratified seeds were sown directly on nutrient-rich soil (Supermix A, Sakata, Japan), and grown in a CO_2_-controlled growth chamber (LPH-0.5P-SH; Nippon Medical & Chemical Instrument) at 280 ppmv or 780 ppmv CO_2_ under 150 µmol m^−2^ s^−1^ fluorescent light (12 h light/12 h dark) at 22 °C. For studies on seedlings, plants were grown on half-strength MS (1/2 MS) agar plates (pH 5.8; 1% agar) placed vertically at 22 °C in the CO_2_-controlled growth chamber at 280 ppmv or 780 ppmv CO_2_ under continuous light (120 µmol m^−2^ s^−1^) unless otherwise noted. To avoid any chamber effects, we used two growth chambers simultaneously with different CO_2_ concentrations and repeated each experiment at least twice with different chamber and CO_2_ concentration combinations. Although the data presented are from one representative experiment, similar results were obtained from different chamber and CO_2_ concentration combinations.

### Quantification of plant hormones

Cytokinin level was determined using an ultra-performance liquid chromatograph coupled with a tandem quadrupole mass spectrometer equipped with an electrospray interface as described previously^75^. IAA and ABA levels were determined using an ultra-high-performance liquid chromatography (UHPLC)-electrospray interface (ESI) and a quadrupole-orbitrap mass spectrometer (UHPLC/Q-Exactive; Thermo Scientific) as described previously^76^. In the results reported, the category iP-type CK precursors comprise iPR and iPRPs; inactivated iP-type CK comprise iP7G and iP9G; tZ-type CK precursors comprise tZR and tZRPs; and inactivated tZ-type CK comprise tZ7G, tZ9G, tZOG, tZROG, and tZRPsOG.

### Gene expression analysis

Total RNA was extracted from root and shoot samples using the RNeasy Plant Mini kit (QIAGEN) in combination with the RNase-Free DNase set (QIAGEN). Total RNA was used for first strand cDNA synthesis by the SuperScript III First-Strand Synthesis System (Life Technologies) with oligo(dT)_20_ primers. Quantitative reverse transcription-PCR (RT-PCR) was performed on a StepOnePlus Real-Time PCR system (Applied Biosystems) with the KAPA SYBR Fast qPCR kit (KAPA Biosystems). *At4g34270* was used as an internal control because this gene has been shown to be one of the most stably expressed genes in *Arabidopsis*^77,78^. Similar results were obtained using other internal control genes (*At1g13320* and A*t2g28390*) as described by Czechowski *et al*.^78^. Primer sets are listed in Supplementary Table S9.

### DCMU and sugar treatment

For 3-(3,4-dichlorophenyl)-1,1-dimethylurea (DCMU) treatment, 8-day-old Col-0 seedlings grown on 1/2 MS agar plates (1% agar) placed vertically under continuous fluorescent light (120 µmol m^−2^ s^−1^) at 22 °C in a CO_2_-controlled growth chamber at 280 ppmv CO_2_ were sprayed with 40 µM DCMU or mock solution (0.05% ethanol) and exposed to 280 ppmv or 780 ppmv CO_2_ under 120 µmol m^−2^ s^−1^ light or in the dark. The DCMU stock solution was 40 mM in 50% ethanol. For DCMU and sucrose co-treatment, seedlings were treated with 40 µM DCMU or mock solution (0.05% ethanol) and then transferred to 1/2 MS agar plates (1% agar) containing 90 mM sucrose. For sugar treatment, seedlings were transferred to 1/2 MS agar plates (1% agar) containing 90 mM of sorbitol, mannitol, sucrose, glucose, or 45 mM sucrose.

### High CO_2_ and sugar treatment under different nitrogen conditions

Wild-type seedlings were pre-grown for 11 days on modified 1/2 MS agar plates (1% agar) containing 10 mM KNO_3_, 10 mM NH_4_Cl or 5 mM NH_4_NO_3_ as the sole nitrogen source in the CO_2_-controlled growth chamber at 280 ppmv. Seedlings grown with 10 mM KNO_3_, 10 mM NH_4_Cl or 5 mM NH_4_NO_3_ were then transferred to new 1/2 MS agar plates (1% agar) containing 10 mM KNO_3_, 10 mM NH_4_Cl or no nitrogen source, respectively. After 24 h incubation at 280 ppmv, seedlings were subjected to high CO_2_ and sugar treatments under the same nitrogen conditions.

### Growth analysis under low or high CO_2_

For growth analysis of soil-grown plants, stratified seeds were sown directly on nutrient-rich soil (Supermix A, Sakata, Japan), and grown in a CO_2_-controlled growth chamber at 280 ppmv or 780 ppmv CO_2_ under 150 µmol m^−2^ s^−1^ fluorescent light (12 h light/12 h dark) at 22 °C and 60% relative humidity. Shoots were harvested at 17 and 31 days after germination (DAG) and their dry weights were determined after drying them in an oven set at 80 °C for three days. Rosette leaf number was counted on 31 DAG.

For seedling growth analysis, surface sterilized seeds were sown on 1/2 MS agar plates (1% agar) containing 1% sucrose. After stratification, plates were placed vertically in a CO_2_-controlled growth chamber (120 µmol m^−2^ s^−1^ continuous fluorescent light, 22 °C) at 280 ppmv. Five-day-old seedlings were transferred to 1/2 MS agar plates (1% agar without sucrose) and grown vertically for another 7 days at 280 ppmv. Then, the 12-day-old seedlings were exposed to 280 ppmv or 780 ppmv CO_2_ for seven days. The shoots and roots were separated and their fresh weights were measured before (Supplementary Figs S4a; S5) and after exposure (Fig. 7c). Relative growth rate (RGR) was calculated from the dry and fresh weights as described elsewhere^79^.

To avoid any chamber effects, we used two growth chambers simultaneously with different CO_2_ concentrations and repeated each experiment at least twice with different chamber and CO_2_ concentration combinations. Although the data presented are from one representative experiment, similar results were obtained from different chamber and CO_2_ concentration combinations.

### Statistical analysis

Data are given as means ± standard error (SE) or means ± standard deviation (SD) of one representative experiment. In order to examine whether hormone concentration, gene expression, or shoot growth were significantly different between treatments, Student’s t-test, two-way ANOVA, and Tukey’s honest significant difference (HSD) test were performed using KaleidaGraph ver. 4.1 software (Synergy Software).

### Accession numbers

Sequence data for the genes described in this article can be found in The Arabidopsis Information Resource database (see http://www.arabidopsis.org) under the following accession numbers: *CYP735A1* (At5g38450), *CYP735A2* (At1g67110), *AtIPT1* (At1g68460), *AtIPT3* (At3g63110), *AtIPT4* (At4g24650) *AtIPT5* (At5g19040), *AtIPT6* (Ay1g25410), *AtIPT7* (At3g23630), *AtIPT8* (At3g19160), *CKX1* (At2g41510), *AtCKX2* (At2g19500), *CKX3* (At5g56970), *CKX4* (At4g29740), *CKX5* (At1g75450), *CKX6* (At3g63440), *CKX7* (At5g21482), *ARR4* (At1g10470), *ARR6* (At5g62920), *ARR15* (At1g74890), *ABCG14* (At1g31770).

## Supplementary information

Supplementary Figures and Tables

## Data Availability

The datasets generated during and/or analysed during the current study are available from the corresponding author on reasonable request.
