# Supplementary material for: Sugar-induced de novo cytokinin biosynthesis contributes to Arabidopsis growth under elevated CO2
Source: Sci Rep. 2019 May 23;9:7765. doi: 10.1038/s41598-019-44185-4 (PMC6533260; doi:10.1038/s41598-019-44185-4)
Supplement: Supplementary file 1 — Supplementary Figures and Tables [file 41598_2019_44185_MOESM1_ESM.pdf]

Sugar-induced *de novo* cytokinin biosynthesis contributes to Arabidopsis growth under elevated CO_2

Takatoshi Kiba[1, 2*], Yumiko Takebayashi[2], Mikiko Kojima[2], and Hitoshi Sakakibara[1, 2*]

1. Department of Applied Biosciences, Graduate School of Bioagricultural Sciences, Nagoya University, Chikusa, Nagoya, 464-8601, Japan
2. RIKEN Center for Sustainable Resource Science, 1-7-22, Suehiro, Tsurumi, Yokohama 230-0045, Japan

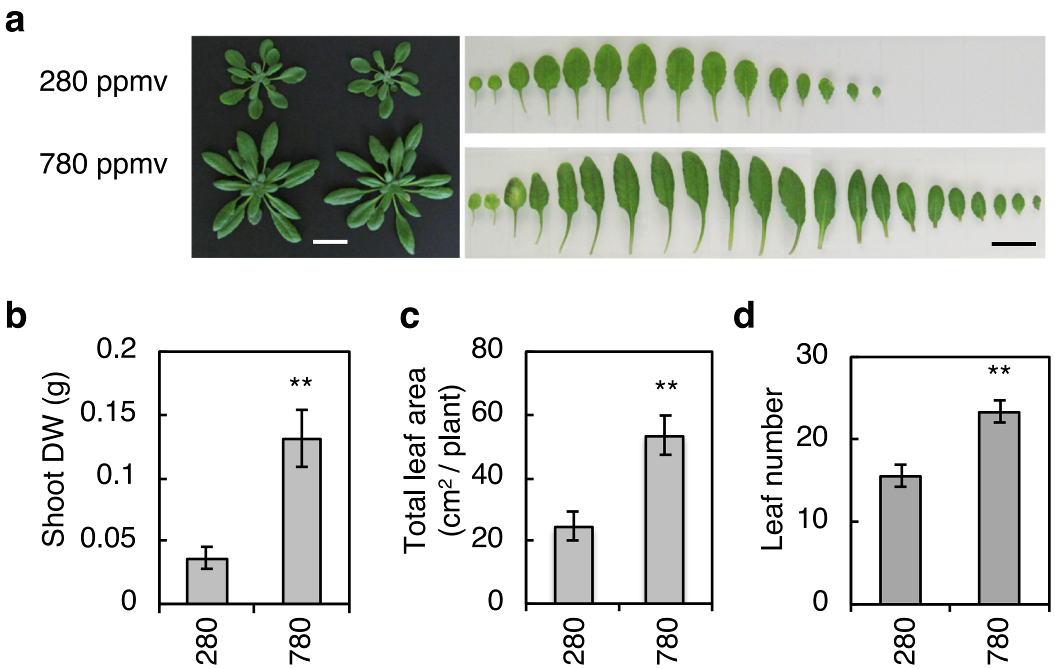

Supplementary Figure S1. Effects of high CO_2 on the growth of soil-grown wild type plants. Representative photographs (a), shoot dry weight (b), total leaf area (c), and leaf number (d) of wild-type Col-0 grown under 280 ppmv CO_2 (280) or 780 ppmv CO_2 (780) for 4 weeks on soil. Error bars represent standard deviation of biological replicates (b, n = 10; c, n = 15; d, n = 5). Asterisks indicate statistically significant differences between 280 ppmv CO_2- and 780 ppmv CO_2-treated samples (**, $p < 0.01$; Student's *t*-test). DW, dry weight. Scale bars, 2.5 cm.

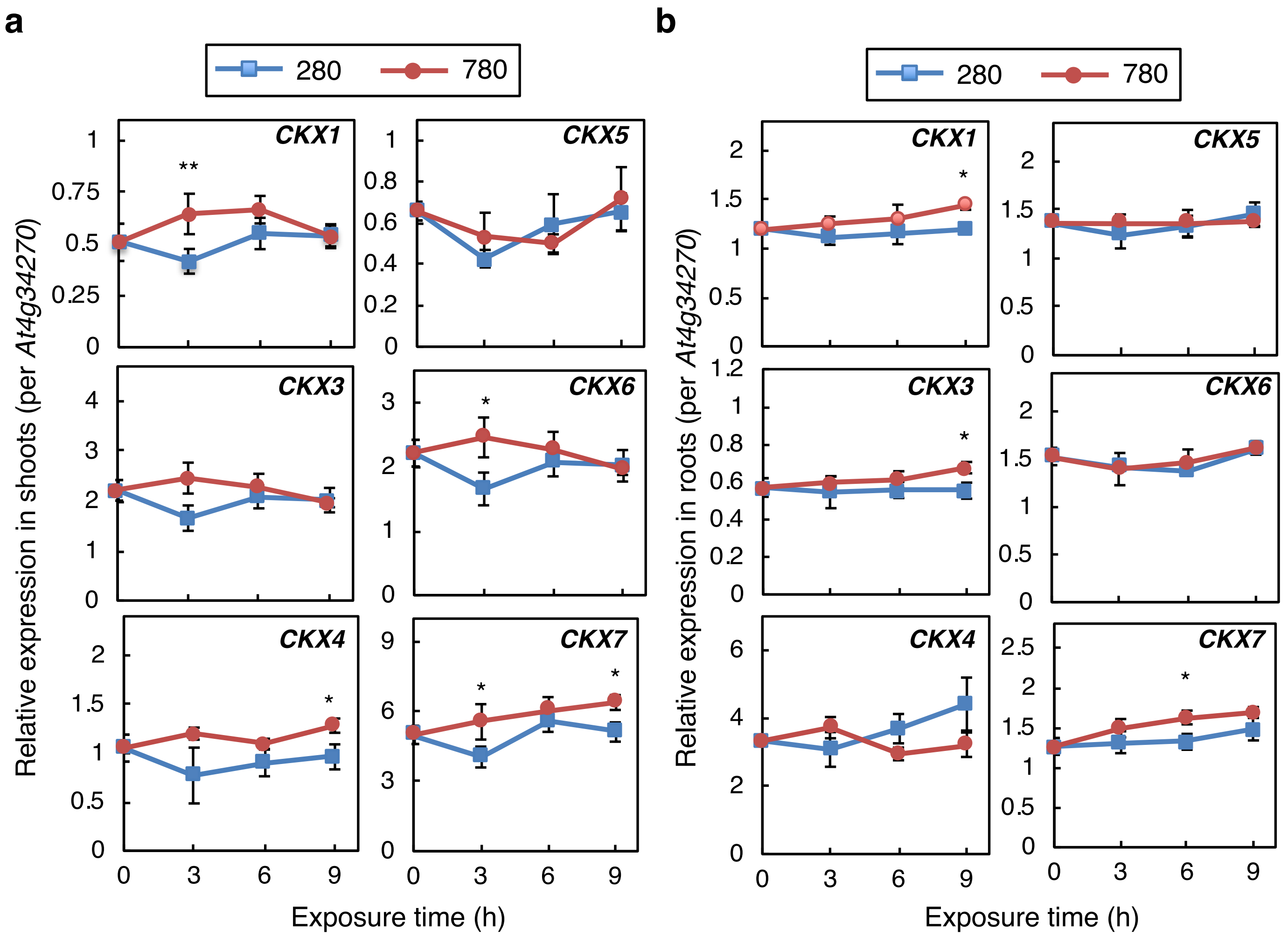

Supplementary Figure S2. Expression of genes involved in cytokinin degradation in shoots and roots upon exposure to high CO_2. Transcript levels of *CKX1*, *CKX2*, *CKX3*, *CKX4*, *CKX5*, *CKX6*, and *CKX7* were analyzed in shoots (a) and roots (b) of wild-type seedlings by quantitative realtime-PCR. Expression levels of *CKX2* were under the detection limit in shoots and roots. Expression levels were normalized using *At4g34270* as an internal control. Twelve day-old seedlings grown on 1/2MS agar plates at 280 ppmv were exposed to 280 ppmv (280) or 780 ppmv (780) CO_2 for the indicated periods. Error bars represent standard deviations of four biological replicates. Asterisks indicate statistically significant differences between 280 ppmv CO_2- and 780 ppmv CO_2-treated samples at the same exposure time (**, $p < 0.01$; *, $p < 0.05$; Student's t-test).

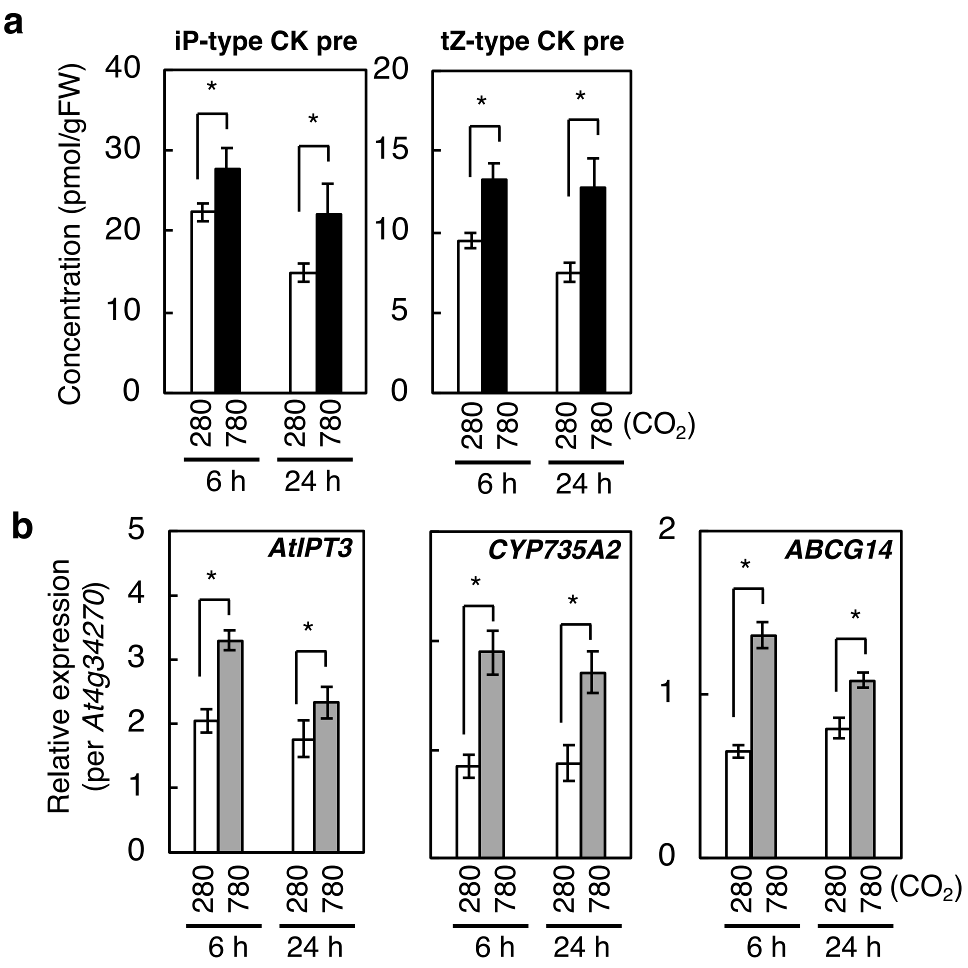

Supplementary Figure S3. Cytokinin levels and expression of cytokinin-related genes in seedlings grown under 12-h-light/12-h-dark conditions and exposed to high CO_2. (**a**) Cytokinin levels in Col-0 whole seedlings exposed to low and high CO_2. iP-type (iP-type CK pre) and tZ-type cytokinin precursor (tZ-type CK pre) levels are presented. (**b**) Expression levels of cytokinin-related genes (*AtIPT3, CYP735A2, ABCG14*) in the roots of Col-0 seedlings exposed to low and high CO_2. Expression levels were normalized using *At4g34270* as an internal control. Twelve-day-old seedlings grown in 1/2 MS agar plates under 12-h-light/12-h-dark conditions at 280 ppmv were exposed to 280 ppmv (280) or 780 ppmv (780) CO_2 for the indicated period. To avoid roots being exposed directly to light, agar plates were wrapped in aluminium foil in a way that only the shoots were directly exposed to light. Exposure to CO_2 commenced when the light was switched on. Error bars represent standard deviations of three biological replicates. Asterisks indicate statistically significant differences between 280 ppmv CO_2- and 780 ppmv CO_2-treated samples at the same exposure time (*, $p < 0.05$, Student's t-test). FW, fresh weight; tZ, trans-zeatin; iP, N^6-(Δ2-isopentenyl)adenine. The concentrations of cytokinin molecular species are shown in Supplementary Table S3.

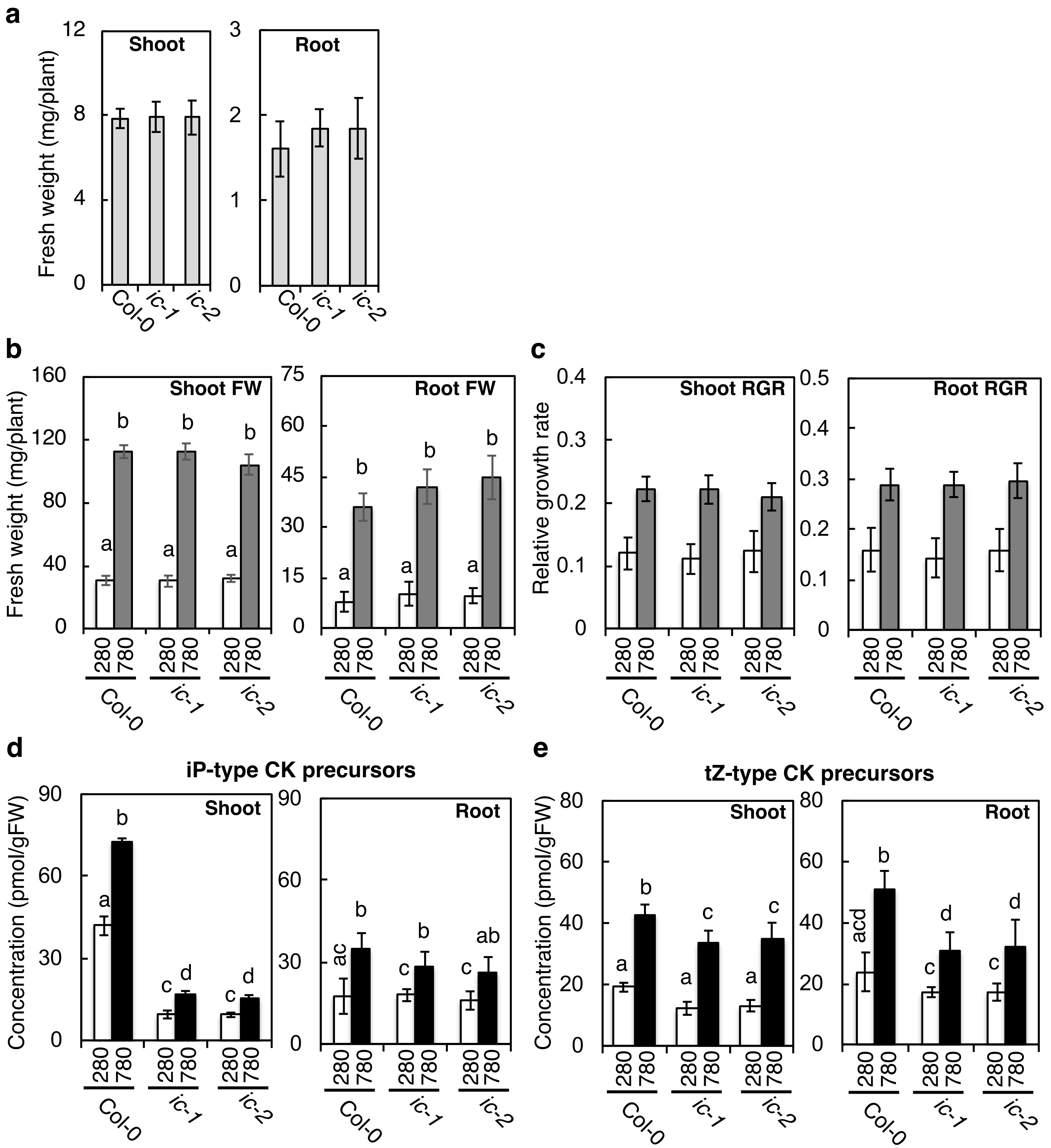

Supplementary Figure S4. Growth parameters and cytokinin levels of *ipt3 cyp735a2* double mutant exposed to high CO_2. (a) Fresh-weight of 12 day-old wild-type (Col-0), *ipt3 cyp735a2-1* (*ic-1*), *and ipt3 cyp735a2-2* (*ic-2*) measured before exposure to high CO_2. This data was used to calculate relative growth rate shown in (c). **(b, c)** Fresh-weight (b) and relative growth rate (RGR) (c) of 19 day-old wild type (Col-0), *ipt3 cyp735a2-1*, and *ipt3 cyp735a2-2* seedlings treated under 280 ppmv (280) or 780 ppmv CO_2 (780) for 7 days. (c) RGR (FW/FW/day) was calculated using fresh weight (FW) data obtained before (a) and after (b) low or high CO_2 treatment. **(d, e)** Cytokinin (CK) levels in shoots and roots of Col-0, *ic-1*, and *ic-2* seedlings exposed to 280 ppmv (280) or 780 ppmv CO_2 (780) for 24h. iP-type CK precursor levels (d) and tZ-type CK precursor levels (e) in shoots and roots are presented. Error bars represent standard deviations (a, n = 9; b, n = 9; d, n = 4; e, n = 4) and standard error (c; n = 9) of biological replicates. Different lower-case letters indicate statistically significant classes (Tukey's HSD test, $p < 0.05$).

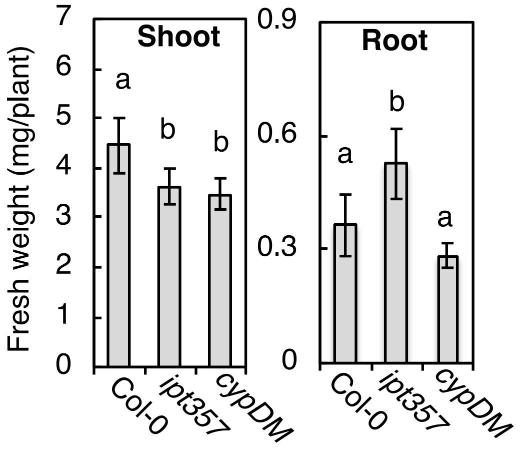

Supplementary Figure S5. Fresh-weight of 12 day-old wild-type*, ipt3 ipt5 ipt7* and *cyp735a1 cyp735a2* measured before exposure to high CO_2. Wild-type (Col-0), *ipt3 ipt5 ipt7* (*ipt357*)and *cyp735a1-2 cyp735a2-2* (*cypDM*) were grown for 12 days and fresh-weight of the shoot and the root was measured. This data was used to calculate relative growth rate shown in Figure 8d. Error bars represent standard deviation of biological replicates (n = 9). Different lower-case letters indicate statistically significant classes (Tukey's HSD test, $p < 0.05$).

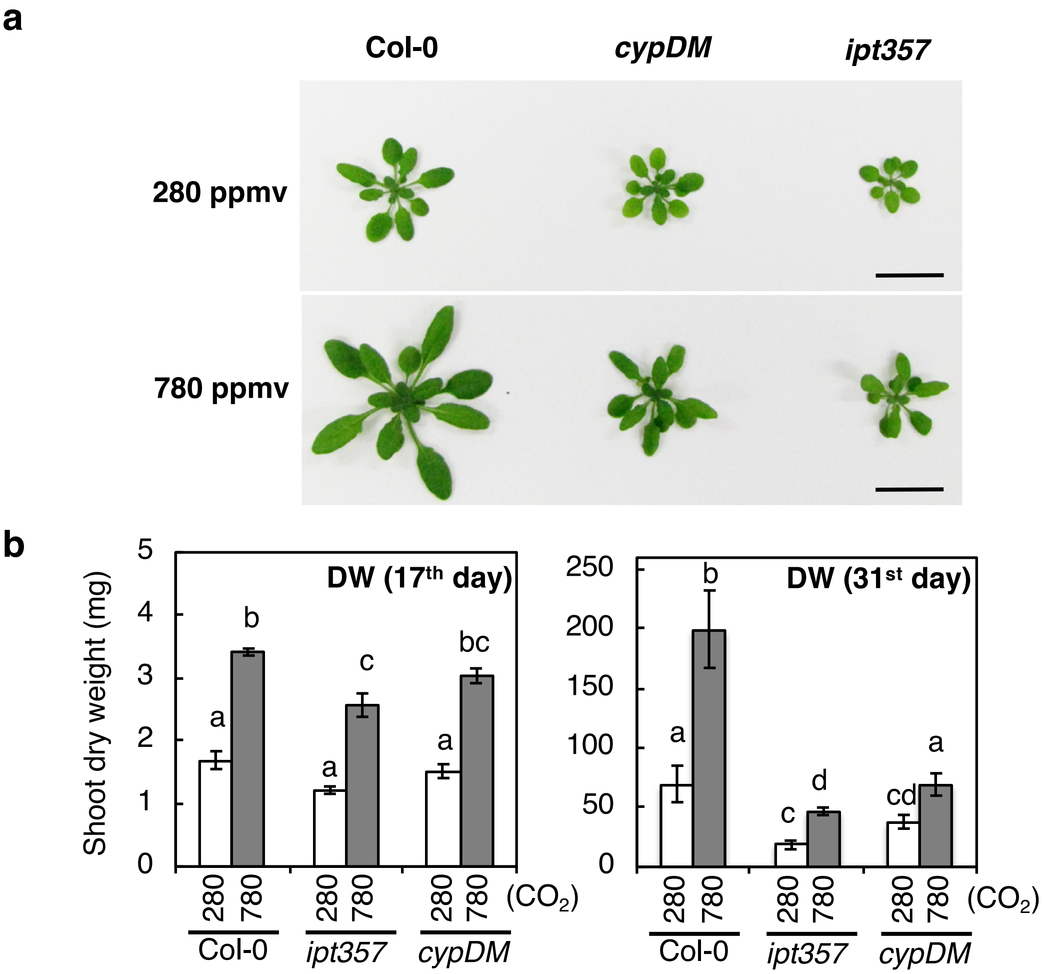

Supplementary Figure S6. Shoot growth of soil-grown wild-type, *ipt3 ipt5 ipt7* and *cyp735a1 cyp735a2* plants under low or high CO_2 (**a**) A representative picture of wild type (Col-0*), cyp735a1 cyp735a2* (*cypDM*) and *ipt3 ipt5 ipt7* (*ipt357*) grown for 24 days at 280 ppmv CO_2 (280) or 780 ppmv CO_2 (780) on soil. Scale bars, 5 cm. (**b**) Dry weight (DW) of Col-0, *cypDM* and *ipt357* aerial parts grown on soil. Dry weights of shoots harvested at 17 and 31 days after germination (DAG) were measured. Error bars represent standard deviations (n = 15). Lower-case letters indicate statistically significant classes (Tukey's HSD test, $p < 0.05$). This data was used to calculate the relative growth rate shown in Figure 8e.

Supplementary Table S1. Hormone concentration in the shoot of wild-type plants exposed to low or high CO_2

Shoots were harvested from soil-grown 3 week-old plants exposed to 280 ppmv and 780 ppmv CO_2 for indicated period. Data are means ± standard deviation (n = 8).

pmol/gFW	0	1 day		2 days		4 days	
	280 ppmv	280 ppmv	780 ppmv	280 ppmv	780 ppmv	280 ppmv	780 ppmv
tZ	5.72±0.7	4.11±0.44	3.75±0.72	2.76±0.33	2.88±0.39	2.34±0.48	3.04±0.42
tZR	5.05±0.6	4.32±0.52	6.02±0.56*	4.47±0.48	6.42±0.38*	4.56±0.65	7.45±1.09*
tZRPs	105.36±12.33	97.15±6.78	127.36±19.19*	98.46±12.58	138.92±11.31*	102.03±14.18	143.59±23.15*
cZ	0.21±0.03	0.2±0.02	0.16±0.03	0.17±0.02	0.13±0.02*	0.18±0.03	0.17±0.02
cZR	0.35±0.06	0.43±0.09	0.24±0.04*	0.44±0.03	0.29±0.11	0.52±0.08	0.52±0.09
cZRPs	2.93±0.34	2.79±0.48	2.12±0.37*	3.1±0.35	3.04±1.63	3.5±0.42	3.97±0.61
DZ	0.06±0.01	0.04±0.03	0.03±0.03	0.05±0.01	0.06±0.03	0.03±0.01	0.05±0.01*
DZR	0.03±0	0.02±0	0.03±0*	0.02±0	0.03±0.01*	0.02±0	0.03±0.01*
DZRPs	0.75±0.11	0.69±0.08	0.76±0.16	0.66±0.1	0.95±0.21*	0.6±0.1	0.96±0.27
iP	0.12±0.01	0.1±0.01	0.14±0.05	0.09±0.01	0.21±0.22	0.09±0.01	0.13±0.01*
iPR	0.14±0.04	0.11±0.03	0.18±0.03*	0.14±0.01	0.22±0.05*	0.15±0.03	0.24±0.03*
iPRPs	47.48±7.1	46.4±6.2	85±11.55*	49.41±4.51	88.87±8.43*	48.23±7.61	93.21±7.8*
tZ7G	154.9±12.61	138.06±14.38	107.67±9.71*	105.01±10.27	95.18±5.39*	95.16±5.65	94.77±10.01
tZ9G	56.41±5.11	56.27±3.35	53.07±3.3	56.35±6.34	54.53±2.83	52.5±3.26	55.8±2.95
tZOG	7.37±1.08	9.26±0.85	9.71±0.79	8.74±0.84	8.6±0.4	9.59±0.5	9.69±0.94
cZOG	N.D.	N.D.	N.D.	N.D.	N.D.	N.D.	N.D.
tZROG	1.24±0.09	1.14±0.06	1.09±0.05	1.16±0.11	1.25±0.08	1.24±0.07	1.51±0.09*
cZROG	1.36±0.09	1.39±0.1	1.25±0.12	1.49±0.09	1.05±0.12*	1.64±0.08	1.24±0.18*
tZRPsOG	0.11±0.06	0.12±0.05	0.07±0.06	0.1±0.05	0.14±0.02	0.14±0.07	0.17±0.05
cZRPsOG	N.D.	N.D.	N.D.	N.D.	N.D.	N.D.	N.D.
DZ9G	0.24±0.02	0.22±0.01	0.2±0.01*	0.2±0.02	0.22±0.01	0.19±0.01	0.24±0.02*
iP7G	17.38±1.15	18.04±0.46	17.23±0.78	16.46±0.7	15.6±0.95	16.62±0.83	15.29±0.82
iP9G	4.05±0.14	3.68±0.13	3.47±0.22	3.21±0.12	3.07±0.16	3.26±0.13	2.96±0.13*
tZ-type CK precursors	110.41±12.93	101.47±7.3	133.38±19.75*	102.93±13.06	145.34±11.69*	106.59±14.83	151.04±24.24*
iP-type CK precursors	47.62±7.14	46.51±6.23	85.18±11.58*	49.55±4.52	89.09±8.48*	48.38±7.64	93.45±7.83*
inactivated tZ-type	220.03±18.95	204.85±18.69	171.61±13.91*	171.36±17.61	151.1±8.32	149.04±9.05	152.25±13.1
inactivated iP-type	21.43±1.29	21.72±0.59	20.7±1	19.67±0.82	18.67±1.11	19.88±0.96	18.25±0.95
GA24	14±0.55	13.42±0.86	12.18±1.05	12.48±0.5	12.05±1.06	11.92±0.98	12.58±0.99
IAA	2191.01±468.72	1607.9±226.32	1694.33±325.48	1715.25±278.82	1760.58±307.51	1823.22±361.19	1530.91±151.01
ABA	17.36±1.61	13.22±1.23	12.51±2.03	14.45±1.77	11.85±1.6	15.42±3.45	15.03±4.13

, statistically significant differences between 280 ppmv CO_2- and 780 ppmv CO_2-treated samples at the same exposure time (, $p < 0.01$; Student's *t*-test).

gFW, gram fresh weight; tZ, *trans*-zeatin; tZR ,tZ riboside; tZRPs, tZ ribotides; cZ, cis-zeatin; cZR, cZ riboside; cZRPs, cZ ribotides; DZ, dihydrozeatin; DZR, DZ riboside; DZRPs, DZ ribotide; iP, N6-(Δ2-isopentenyl)adenine; iPR, iP riboside; iPRPs, iP ribotides; tZ7G, tZ-7-N-glucoside; tZ9G, tZ-9-N-glucoside; tZOG, tZ-O-glucoside; cZOG, cZ-O-glucoside; tZROG, tZR-O-glucoside; cZROG, cZR-O-glucoside; DZ9G, DZ-9-N-glucoside; iP7G, iP-7-N-glucoside; iP9G, iP-9-N-glucoside; GA, gibberellin; IAA, indole-3-acetic acid; ABA, abscisic acid; tZ-type CK precursors, sum of tZR and tZRPs; iP-type CK precursors, sum of iPR and iPRPs; inactivated tZ-type, sum of tZ7G, tZ9G, tZOG, tZROG and tZRPsOG; inactivated iP-type, sum of iP7G and iP9G; N.D., not detected.

Supplementary Table S2. Cytokinin concentration in shoots and roots of wild-type seedlings grown and exposed to low or high CO_2

Shoots and roots were harvested from 12 day-old seedlings grown on 1/2MS agar plates under continuous light at 280 ppmv and then exposed to 280 ppmv or 780 ppmv CO_2 for the indicated periods. Data are means ± standard deviation (n = 3).

pmol/gFW	6 h exposure				24 h exposure			
	Shoot		Root		Shoot		Root	
	280 ppmv	780 ppmv	280 ppmv	780 ppmv	280 ppmv	780 ppmv	280 ppmv	780 ppmv
tZ	0.38±0.03	0.54±0.01*	1.54±0.08	2.43±0.12*	0.52±0.05	0.57±0.02*	1.51±0.23	2.11±0.1*
tZR	0.2±0.04	0.45±0.02*	2.55±0.65	5.49±0.14*	0.44±0.03	0.6±0.06*	3.37±0.91	5.49±0.38*
tZRPs	2.18±0.35	4.19±0.52*	4.24±0.11	7.96±0.38*	3.82±0.27	6.03±0.37*	3.23±0.65	7.81±0.25*
cZ	N.D.	N.D.	N.D.	N.D.	N.D.	N.D.	N.D.	N.D.
cZR	0.24±0.03	0.09±0.07	1.91±0.85	1.65±0.39	0.21±0.01	0.18±0.1	5.56±0.57	6.32±0.79
cZRPs	1.42±0.05	0.7±0.24*	5.43±0.28	5.03±1.03	1.04±0.06	0.98±0.27	9.77±1.11	11.74±0.72
DZ	N.D.	N.D.	N.D.	N.D.	N.D.	N.D.	N.D.	N.D.
DZR	N.D.	N.D.	N.D.	N.D.	N.D.	N.D.	N.D.	N.D.
DZRPs	N.D.	N.D.	N.D.	N.D.	N.D.	N.D.	N.D.	N.D.
iP	0.05±0	0.05±0	0.15±0.01	0.16±0.01	0.05±0	0.04±0*	0.3±0.01	0.24±0.02*
iPR	0.01±0	0.02±0*	0.11±0.01	0.16±0.01*	0.01±0	0.02±0*	0.15±0.02	0.18±0.01
iPRPs	11.39±1.18	13.72±1.27*	5.22±0.96	6.83±0.03*	10.17±0.41	13.97±0.74*	5.28±0.62	8.5±0.34*
tZ7G	14.17±0.61	12.96±0.56	23.03±1.36	26.79±1.52	12.99±0.63	13.45±0.69	29.9±1.64	28.37±0.67
tZ9G	7.12±0.38	6.47±0.37	15.85±0.24	18.72±0.81*	6.32±0.14	7.31±0.45	20.79±0.81	21.67±0.93
tZOG	4.19±0.4	4.75±0.12	5.14±0.47	6.64±0.18	4.64±0.3	4.34±0.26	6.63±0.63	5.49±0.76
cZOG	N.D.	N.D.	N.D.	N.D.	N.D.	N.D.	N.D.	N.D.
tZROG	0.12±0.01	0.12±0.01	0.07±0	0.08±0.01	0.11±0.01	0.11±0	0.07±0.01	0.08±0
cZROG	1.44±0.06	1.32±0.11	0.15±0.01	0.14±0.01	1.42±0.13	1.16±0.08*	0.18±0.02	0.14±0.02
tZRPsOG	N.D.	N.D.	N.D.	N.D.	N.D.	N.D.	N.D.	N.D.
cZRPsOG	N.D.	N.D.	N.D.	N.D.	N.D.	N.D.	N.D.	N.D.
DZ9G	0.11±0	0.1±0	0.15±0.01	0.17±0.01	0.1±0.01	0.09±0.01	0.19±0.01	0.18±0.01
iP7G	14.19±0.29	14.2±0.73	6.31±0.04	6.83±0.21	14.18±0.45	11.28±0.5*	7.4±0.24	6.42±0.29*
iP9G	3.1±0.06	3.11±0.16	1.15±0.04	1.31±0.05	3.03±0.06	2.39±0.12*	1.4±0.06	1.1±0.1
iP-type CK precursors	11.4±1.18	13.74±1.27*	5.33±0.97	6.99±0.04*	10.18±0.41	13.99±0.74*	5.43±0.64	8.68±0.35*
tZ-type CK precursors	2.38±0.39	4.64±0.54*	6.79±0.76	13.45±0.52*	4.26±0.3	6.63±0.43*	6.60±1.56	13.30±0.63*

, statistically significant differences between 280 ppmv CO_2- and 780 ppmv CO_2-treated samples at the same exposure time (, $p < 0.01$; Student's *t*-test).

gFW, gram fresh weight; tZ, *trans*-zeatin; tZR ,tZ riboside; tZRPs, tZ ribotides; cZ, cis-zeatin; cZR, cZ riboside; cZRPs, cZ ribotides; DZ, dihydrozeatin; DZR, DZ riboside; DZRPs, DZ ribotide; iP, N6-(Δ2-isopentenyl)adenine; iPR, iP riboside; iPRPs, iP ribotides; tZ7G, tZ-7-N-glucoside; tZ9G, tZ-9-N-glucoside; tZOG, tZ-O-glucoside; cZOG, cZ-O-glucoside; tZROG, tZR-O-glucoside; cZROG, cZR-O-glucoside; DZ9G, DZ-9-N-glucoside; iP7G, iP-7-N-glucoside; iP9G, iP-9-N-glucoside; tZ-type CK precursors, sum of tZR and tZRPs; iP-type CK precursors, sum of iPR and iPRPs; N.D., not detected.

Supplementary Table S3. Cytokinin concentration in wild-type seedlings grown and exposed to low or high CO_2 under 12-h-light/12-h-dark cycles

Twelve-day-old seedlings grown in 1/2 MS agar plates under 12-h-light/12-h-dark conditions at 280 ppmv were exposed to 280 ppmv (280) or 780 ppmv (780) CO_2 for the indicated period. To avoid roots being exposed directly to light, agar plates were wrapped in aluminium foil in a way that only the shoots were directly exposed to light. Exposure to CO_2 commenced when the light was switched on. Error bars represent standard deviations of four biological replicates.

pmol/gFW	6h exposure		24h exposure	
	280 ppmv	780 ppmv	280 ppmv	780 ppmv
tZ	0.32±0.03	0.41±0.04*	0.3±0.03	0.35±0.05
tZR	0.58±0.02	0.75±0.07*	0.51±0.12	0.82±0.19*
tZRPs	8.57±0.51	12.03±1.02*	6.68±0.5	11.61±1.82*
cZ	0.12±0.01	0.1±0.01	0.13±0.01	0.11±0.03
cZR	0.23±0.02	0.2±0.03	0.5±0.06	0.38±0.05*
cZRPs	3.93±0.41	3.51±0.19	5.84±0.28	4.84±0.73
DZ	N.D	N.D	N.D	N.D
DZR	N.D	N.D	N.D	N.D
DZRPs	0.21±0.03	0.24±0.04	0.15±0.02	0.22±0.02*
iP	0.26±0.02	0.31±0.02*	0.22±0.02	0.25±0.04
iPR	0.31±0.05	0.44±0.1*	0.3±0.07	0.5±0.22
iPRPs	21.8±1.12	26.95±2.57*	14.39±1.07	21.3±4.02*
tZ7G	15.51±0.87	17.04±0.58*	15.84±0.48	17.28±0.39*
tZ9G	4.13±0.25	4.23±0.07	4.09±0.15	4.51±0.26*
tZOG	5.44±0.36	5.26±0.62	5.64±0.5	5.03±0.36
cZOG	1.69±0.15	1.92±0.4	2.26±0.54	1.49±0.35
tZROG	0.35±0.03	0.35±0.02	0.3±0.02	0.36±0.08
cZROG	1.53±0.17	1.89±0.42	2.09±0.27	1.46±0.4
tZRPsOG	N.D	N.D	N.D	N.D
cZRPsOG	N.D	N.D	N.D	N.D
DZ9G	0.13±0.01	0.13±0.02	0.14±0.01	0.12±0.01
iP7G	149.05±16.52	157.34±6.43	169.45±11.8	143.56±9.54*
iP9G	2.63±0.1	2.9±0.13*	3.09±0.17	2.68±0.12*
iP-type CK precursors	9.15±0.53	12.78±1.09*	7.19±0.62	12.43±2.01*
tZ-type CK precursors	22.1±1.17	27.39±2.68*	14.69±1.14	21.8±4.24*

, statistically significant differences between 280 ppmv CO_2- and 780 ppmv CO_2-treated samples at the same exposure time (, $p < 0.01$; Student's *t*-test).

gFW, gram fresh weight; tZ, *trans*-zeatin; tZR ,tZ riboside; tZRPs, tZ ribotides; cZ, cis-zeatin; cZR, cZ riboside; cZRPs, cZ ribotides; DZ, dihydrozeatin; DZR, DZ riboside; DZRPs, DZ ribotide; iP, N6-(Δ2-isopentenyl)adenine; iPR, iP riboside; iPRPs, iP ribotides; tZ7G, tZ-7-N-glucoside; tZ9G, tZ-9-N-glucoside; tZOG, tZ-O-glucoside; cZOG, cZ-O-glucoside; tZROG, tZR-O-glucoside; cZROG, cZR-O-glucoside; DZ9G, DZ-9-N-glucoside; iP7G, iP-7-N-glucoside; iP9G, iP-9-N-glucoside; tZ-type CK precursors, sum of tZR and tZRPs; iP-type CK precursors, sum of iPR and iPRPs; N.D., not detected.

Supplementary Table S4. Cytokinin concentration in shoots and roots of wild-type seedlings treated with sucrose

Shoots and roots were harvested from 12-day-old seedlings grown on 1/2MS agar plates at 280 ppmv treated with 45 mM sucrose (+Suc) or without sucrose (-Suc) for 24h. Data are means ± standard deviation (n = 4).

pmol/gFW	24 h-treatment			
	Shoot		Root	
	-Suc	+Suc	-Suc	+Suc
tZ	0.15±0.03	0.20±0.01*	1.72±0.19	2.05±0.28
tZR	0.13±0.03	0.23±0.05*	3.28±0.37	5.01±1.00*
tZRPs	2.93±0.46	5.16±0.36*	10.08±1.33	14.39±2.6*
cZ	0.17±0.02	0.11±0.06	0.95±0.25	0.58±0.54
cZR	0.32±0.02	0.25±0.03	3.36±0.64	3.41±0.52
cZRPs	4.65±0.14	3.72±0.16	12.43±1.69	12.66±1.71
DZ	N.D.	N.D.	N.D.	N.D.
DZR	N.D.	N.D.	N.D.	N.D.
DZRPs	0.11±0.02	0.16±0.05*	N.D.	N.D.
iP	0.22±0.14	0.14±0.13	0±0	0.91±2.05
iPR	0.13±0.04	0.17±0.08	0.36±0.35	0.68±0.46
iPRPs	13.98±0.89	19.04±1.35*	7.63±0.55	9.87±1.62*
tZ7G	10.80±0.60	11.07±0.47	39.50±2.97	43.70±4.28
tZ9G	3.04±0.29	3.05±0.19	16.31±1.11	17.4±1.7
tZOG	3.90±0.27	3.71±0.29	21.14±1.82	22.91±2.95
cZOG	1.60±0.21	1.43±0.21	7.11±0.88	6.46±0.63
tZROG	0.38±0.03	0.38±0.04	N.D.	N.D.
cZROG	1.65±0.12	1.62±0.12	1.27±0.18	0.96±0.09*
tZRPsOG	N.D.	N.D.	N.D.	N.D.
cZRPsOG	N.D.	N.D.	N.D.	N.D.
DZ9G	0.08±0.01	0.08±0	N.D.	N.D.
iP7G	174.55±30.57	150.08±29.57	71.5±5.34	76.26±7.96
iP9G	2.75±0.07	2.71±0.11	1.31±0.13	1.33±0.21
iP-type CK precursors	14.11±0.93	19.21±1.44*	7.99±0.90	10.55±2.08*
tZ-type CK precursors	3.07±0.49	5.39±0.40*	13.36±1.70	19.40±3.60*

, statistically significant increase in +Suc compared with -Suc (, $p < 0.01$; Student's *t*-test).

gFW, gram fresh weight; tZ, *trans*-zeatin; tZR ,tZ riboside; tZRPs, tZ ribotides; cZ, cis-zeatin; cZR, cZ riboside; cZRPs, cZ ribotides; DZ, dihydrozeatin; DZR, DZ riboside; DZRPs, DZ ribotide; iP, N6-(Δ2-isopentenyl)adenine; iPR, iP riboside; iPRPs, iP ribotides; tZ7G, tZ-7-N-glucoside; tZ9G, tZ-9-N-glucoside; tZOG, tZ-O-glucoside; cZOG, cZ-O-glucoside; tZROG, tZR-O-glucoside; cZROG, cZR-O-glucoside; DZ9G, DZ-9-N-glucoside; iP7G, iP-7-N-glucoside; iP9G, iP-9-N-glucoside; tZ-type CK precursors, sum of tZR and tZRPs; iP-type CK precursors, sum of iPR and iPRPs; N.D., not detected.

Supplementary Table S5. Cytokinin concentration in wild-type seedlings exposed to low or high CO_2 under different nitrogen nutrient conditions

Twelve day-old seedlings were exposed to 280 ppmv or 780 ppmv CO_2 for 24h on 1/2MS agar plates with different nitrogen conditions. Nitrogen conditions were 10 mM KNO_3 (NO_3^-), 10 mM NH_4Cl, (NH_4^+) and no nitorogen (-N). Data are means ± standard deviation (n = 4).

pmol/gFW	NO_3^-		NH_4^+		-N	
	280 ppmv	780 ppmv	280 ppmv	780 ppmv	280 ppmv	780 ppmv
tZ	0.23±0.03	0.25±0.03	0.38±0.07	0.52±0.06*	0.17±0.01	0.16±0.02
tZR	0.28±0.02	0.38±0.04*	0.46±0.03	0.65±0.09*	0.19±0.01	0.31±0.05*
tZRPs	4.5±0.45	6.86±0.56*	7.95±0.87	11.48±1.21*	3.16±0.21	3.66±0.14*
cZ	0.13±0.01*	0.09±0.02	0.21±0.03	0.19±0.02	0.22±0.03	0.17±0.02
cZR	0.2±0.02	0.16±0.03	0.24±0.01	0.19±0.03	0.52±0.04	0.48±0.03
cZRPs	3.34±0.19	2.89±0.19*	3.24±0.57	3.16±0.38	7.13±0.72	6.23±0.77
DZ	N.D.	N.D.	N.D.	N.D.	N.D.	N.D.
DZR	N.D.	N.D.	N.D.	N.D.	N.D.	N.D.
DZRPs	N.D.	0.14±0.01	0.14±0.01	0.26±0.05*	N.D.	0.13±0.01
iP	0.21±0.06	0.19±0.01	0.22±0.04	0.24±0	0.22±0.02	0.24±0.03
iPR	0.1±0.02	0.1±0.02	0.12±0.02	0.13±0.01	0.06±0.01	0.1±0.02*
iPRPs	8.23±0.61	12.73±0.63*	12.28±0.82	13.9±0.92*	7.09±0.42	8.1±0.62*
tZ7G	11.53±0.69	11.43±0.46	16.6±0.77	16.74±0.38	15.77±0.74	15.21±1.02
tZ9G	2.91±0.09	2.76±0.14	3.81±0.28	4.02±0.2	3.6±0.18	3.58±0.24
tZOG	4.94±0.34	4.26±0.5	4.74±0.28	5.07±0.36	5.43±0.49	4.8±0.56
cZOG	2.32±0.19	1.84±0.24*	2.35±0.38	2.03±0.09	2.54±0.12	2.14±0.4
tZROG	0.18±0.02	0.17±0.01	0.29±0.06	0.31±0.03	0.3±0.05	0.28±0.02
cZROG	1.39±0.14	1.19±0.15	1.46±0.27	1.22±0.18	2.39±0.19	2.1±0.22
tZRPsOG	N.D.	N.D.	N.D.	N.D.	N.D.	N.D.
cZRPsOG	N.D.	N.D.	N.D.	N.D.	N.D.	N.D.
DZ9G	0.08±0.01	0.08±0.01	0.12±0.01	0.12±0.01	0.14±0.01	0.13±0.02
iP7G	114.46±5.32	111.4±7.61	168.49±14.38	164.29±6.68	182.95±7	164.18±9.35*
iP9G	1.68±0.06	1.6±0.1	2.74±0.15	2.71±0.05	2.83±0.11	2.66±0.13
iP-type CK precursors	4.78±0.47	7.24±0.6*	8.41±0.89	12.13±1.31*	3.35±0.22	3.97±0.19*
tZ-type CK precursors	8.34±0.63	12.83±0.66*	12.4±0.84	14.03±0.94*	7.16±0.43	8.2±0.64*

, statistically significant differences between 280 ppmv CO_2- and 780 ppmv CO_2-treated samples at the same exposure time (, $p < 0.01$; Student’s *t*-test).

gFW, gram fresh weight; tZ, *trans*-zeatin; tZR ,tZ riboside; tZRPs, tZ ribotides; cZ, cis-zeatin; cZR, cZ riboside; cZRPs, cZ ribotides; DZ, dihydrozeatin; DZR, DZ riboside; DZRPs, DZ ribotide; iP, N6-(Δ2-isopentenyl)adenine; iPR, iP riboside; iPRPs, iP ribotides; tZ7G, tZ-7-N-glucoside; tZ9G, tZ-9-N-glucoside; tZOG, tZ-O-glucoside; cZOG, cZ-O-glucoside; tZROG, tZR-O-glucoside; cZROG, cZR-O-glucoside; DZ9G, DZ-9-N-glucoside; iP7G, iP-7-N-glucoside; iP9G, iP-9-N-glucoside; tZ-type CK precursors, sum of tZR and tZRPs; iP-type CK precursors, sum of iPR and iPRPs; N.D., not detected.

Supplementary Table S6. Cytokinin concentration in wild-type seedlings treated with sucrose under different nitrogen nutrient conditions

Twelve day-old seedlings were treated with 45 mM sucrose (+Suc) or without sucrosse (-Suc) for 24h on 1/2MS agar plates of different nitrogen conditions. Nitrogen conditions were 10 mM KNO_3 (NO_3^-), 10 mM NH_4Cl, (NH_4^+) and no nitorogen (-N). Data are means ± standard deviation (n = 4).

pmol/gFW	NO_3^-		NH_4^+		-N	
	-Suc	+Suc	-Suc	+Suc	-Suc	+Suc
tZ	0.23±0.02	0.48±0.04*	0.32±0.03	0.47±0.03*	0.17±0.01	0.32±0.02*
tZR	0.28±0.04	0.55±0.06*	0.31±0.01	0.68±0.01*	0.19±0.01	0.53±0.08*
tZRPs	3.12±0.13	10.55±0.91*	4.76±0.47	10.38±0.38*	3.16±0.21	9.38±1.01*
cZ	0.15±0.04	0.15±0.01	0.14±0.01	0.19±0.01*	0.22±0.03	0.17±0.02
cZR	0.22±0.02	0.29±0.06	0.23±0.04	0.29±0.03	0.52±0.04	0.5±0.07
cZRPs	3.11±0.14	3.26±0.09	3.93±0.33	4.12±0.28	7.13±0.72	6.33±0.49
DZ	N.D.	N.D.	N.D.	N.D.	N.D.	N.D.
DZR	N.D.	N.D.	N.D.	N.D.	N.D.	N.D.
DZRPs	N.D.	0.25±0.07	N.D.	0.24±0.05	N.D.	0.25±0.04
iP	0.2±0.02	0.2±0.02	0.25±0.05	0.22±0.04	0.22±0.02	0.22±0.03
iPR	0.1±0.02	0.16±0.04*	0.08±0.02	0.18±0.02*	0.06±0.01	0.11±0.04
iPRPs	7.83±0.54	13.54±0.86*	11.55±0.78	14.72±0.53*	7.09±0.42	9.47±0.88*
tZ7G	11.15±0.43	12.49±0.27*	15.51±0.8	17.07±0.66	15.77±0.74	17.27±0.71
tZ9G	2.68±0.09	3.03±0.08*	3.52±0.09	4.32±0.13*	3.6±0.18	4.14±0.24*
tZOG	4.37±0.32	4.89±0.35	4.51±0.58	5.42±0.42	5.43±0.49	5.78±0.61
cZOG	2.26±0.18	2.68±0.34	2.3±0.32	2.63±0.17	2.54±0.12	2.22±0.23
tZROG	0.21±0.02	0.23±0.01	0.29±0.01	0.34±0.03	0.3±0.05	0.29±0.02
cZROG	1.44±0.1	1.58±0.05	1.47±0.22	1.71±0.32	2.39±0.19	2.4±0.08
tZRPsOG	N.D.	N.D.	N.D.	N.D.	N.D.	N.D.
cZRPsOG	N.D.	N.D.	N.D.	N.D.	N.D.	N.D.
DZ9G	0.08±0.01	0.1±0.01	0.11±0.01	0.16±0.03	0.14±0.01	0.17±0.02
iP7G	109.04±3.78	122.55±8.36	152.28±7.91	163.38±5.85	182.95±7	179.59±4.85
iP9G	1.68±0.03	1.85±0.08*	2.55±0.12	2.7±0.03	2.83±0.11	2.66±0.09
iP-type CK precursors	3.40±0.17	11.11±0.97*	5.07±0.48	11.07±0.39*	3.35±0.22	9.91±1.09*
tZ-type CK precursors	7.93±0.55	13.71±0.9*	11.63±0.81	14.91±0.55*	7.16±0.43	9.58±0.92*

, statistically significant differences between +Suc and -Suc (, $p < 0.01$; Student's *t*-test).

gFW, gram fresh weight; tZ, *trans*-zeatin; tZR ,tZ riboside; tZRPs, tZ ribotides; cZ, cis-zeatin; cZR, cZ riboside; cZRPs, cZ ribotides; DZ, dihydrozeatin; DZR, DZ riboside; DZRPs, DZ ribotide; iP, N6-(Δ2-isopentenyl)adenine; iPR, iP riboside; iPRPs, iP ribotides; tZ7G, tZ-7-N-glucoside; tZ9G, tZ-9-N-glucoside; tZOG, tZ-O-glucoside; cZOG, cZ-O-glucoside; tZROG, tZR-O-glucoside; cZROG, cZR-O-glucoside; DZ9G, DZ-9-N-glucoside; iP7G, iP-7-N-glucoside; iP9G, iP-9-N-glucoside; tZ-type CK precursors, sum of tZR and tZRPs; iP-type CK precursors, sum of iPR and iPRPs; N.D., not detected.

Supplementary Table S7. Cytokinin concentration in shoots and roots of Col-0, *ipt3 cyp735a2-1* and *ipt3 cyp735a2-2* seedlings exposed to low or high CO_2

Shoots and roots were harvested from 12 day-old seedlings grown on 1/2MS agar plates at 280 ppmv and then exposed to 280 ppmv or 780 ppmv CO_2 for 24h. Data are means ± standard deviation (n = 4).

pmol/gFW	Col-0				*ipt3 cyp735a2-1*				*ipt3 cyp735a2-2*			
	Shoot		Root		Shoot		Root		Shoot		Root	
	280 ppmv	780 ppmv	280 ppmv	780 ppmv	280 ppmv	780 ppmv	280 ppmv	780 ppmv	280 ppmv	780 ppmv	280 ppmv	780 ppmv
tZ	0.43±0.04	0.68±0.04*	2.1±0.43	3.68±0.33*	0.26±0.06	0.5±0.06*	1.37±0.24	1.92±0.28*	0.22±0.05	0.49±0.06*	1.25±0.18	2.07±0.5*
tZR	1.03±0.11	2.39±0.21*	4.69±2.26	11.27±1.87*	0.71±0.21	1.74±0.37*	5.49±0.63	6.66±1.52	0.64±0.05	1.58±0.35*	4.17±0.87	6.66±1.97*
tZRPs	18.1±1.37	40.44±3.15*	19.14±4.25	39.57±4.35*	11.54±1.93	32.1±3.46*	11.67±1.54	24.2±4.46*	12.46±1.86	33.03±5.28*	13.01±2.09	25.43±6.92*
cZ	0.09±0.05	N.D.	0.61±0.05	0.35±0.24	0.08±0.05	N.D.	0.77±0.14	0.37±0.22*	0.07±0.05	0.06±0.05	0.83±0.49	0.54±0.16
cZR	0.15±0.03	0.09±0*	0.89±0.3	1.02±0.1	0.18±0.04	0.07±0.01*	0.94±0.11	0.81±0.25	0.18±0.09	0.09±0.02*	2.72±2.5	0.88±0.27
cZRPs	2.19±0.38	1.66±0.14*	4.85±2.12	6.55±1.17	2.22±0.51	1.3±0.14*	6.87±1.59	5.54±1.36	2.6±1.72	1.38±0.13	6.75±1.44	5.88±1.82
DZ	N.D.	0.06±0	N.D.	N.D.	0.04±0	0.06±0	N.D.	N.D.	N.D.	0.04±0	N.D.	N.D.
DZR	N.D.	N.D.	N.D.	N.D.	N.D.	N.D.	N.D.	N.D.	N.D.	N.D.	N.D.	N.D.
DZRPs	0.4±0.04	1.22±0.14*	0.4±0.11	0.92±0.17*	0.3±0.04	0.83±0.08*	N.D.±N.D.	0.35±0.07	0.35±0.11	0.8±0.1*	0.29±0.07	0.43±0.16
iP	0.25±0.05	0.25±0.05	0.43±0.51	1.25±0.38*	N.D.	N.D.	0.85±0.59	1.24±0.39	0.06±0.1	0.3±0.57	1.36±0.63	1.32±0.35
iPR	0.46±0.18	0.53±0.05	0.83±0.48	1.51±0.42*	0.08±0.01	0.06±0.01	1.43±0.39	1.28±0.32	0.05±0.03	0.04±0.03	0.82±0.36	1.07±0.35
iPRPs	41.43±3.28	72.06±1.27*	16.67±6.02	33.41±5.37*	9.41±1.45	16.64±1.37*	16.45±1.86	27.26±4.95*	9.3±0.83	15.28±1.26*	15.1±3.04	25.05±5.42*
tZ7G	13.71±0.56	13.21±0.52	23.43±3.47	32.24±1.64*	8.6±0.34	8.09±0.22	17.98±0.93	20.65±2.01*	8.98±0.96	8.63±0.18	17.87±2.2	21.32±3.9
tZ9G	4.32±0.23	4.22±0.15	9.28±1.68	13.73±0.95*	2.29±0.11	2.32±0.14	6.53±0.58	7.88±0.62*	2.57±0.5	2.44±0.12	6.73±0.73	8.3±1.55
tZOG	6.18±0.17	6.66±0.25*	16.02±1.73	19.67±2.12*	5.06±0.13	5.38±0.36*	14.97±0.84	15.75±1.56	4.77±0.61	5.02±0.68	15.21±1.69	15.86±2.86
cZOG	1.09±0.1	0.85±0.05*	3.84±0.24	3.97±0.2	1.27±0.1	1.04±0.16*	4.36±0.29	4.11±0.61	1.13±0.26	0.96±0.1	4.42±0.24	4.18±0.44
tZROG	0.56±0.04	0.59±0.02	0.23±0.2	0.1±0.2	0.46±0.03	0.5±0.03	0.27±0.15	0.13±0.18	0.47±0.07	0.5±0.04	0.16±0.18	0.16±0.22
cZROG	1.25±0.09	1.07±0.04*	0.53±0.07	0.52±0.07	1.4±0.13	1.14±0.12*	0.61±0.06	0.58±0.12	1.24±0.17	1.1±0.1	0.6±0.07	0.56±0.08
tZRPsOG	N.D.	N.D.	N.D.	N.D.	N.D.	N.D.	N.D.	N.D.	N.D.	N.D.	N.D.	N.D.
cZRPsOG	N.D.	N.D.	N.D.	N.D.	N.D.	N.D.	N.D.	N.D.	N.D.	N.D.	N.D.	N.D.
DZ9G	0.1±0.01	0.13±0.01	0.75±0.16	1.02±0.07*	0.07±0	0.07±0.01	0.81±0.17	0.83±0.1	0.07±0.02	0.08±0.01	0.8±0.22	N.D.
iP7G	81.58±5.14	72.69±5.11*	50.66±5.15	58.07±2.48*	36.93±0.86	34.76±3*	53.27±2.77	54.92±3.18	34.96±3.05	33.7±2.27	52.47±3.98	57.47±10.41
iP9G	1.57±0.04	1.45±0.12	0.86±0.09	1.06±0.06*	0.63±0.02	0.59±0.05	1.08±0.04	1.05±0.09	0.6±0.06	0.61±0.05	1.05±0.1	1.12±0.2
iP-type CK precursor	41.89±3.46	72.59±1.32*	17.5±6.5	34.92±5.79*	9.49±1.46	16.7±1.38*	17.88±2.25	28.54±5.27*	9.35±0.86	15.32±1.29*	15.92±3.4	26.12±5.77*
tZ-type CK precursor	19.13±1.48	42.83±3.36*	23.83±6.51	50.84±6.22*	12.25±2.14	33.84±3.83*	17.16±2.17	30.86±5.98*	13.1±1.91	34.61±5.63*	17.18±2.96	32.09±8.89*

, statistically significant differences between 280 ppmv CO2- and 780 ppmv CO_2-treated samples of the same genotype (, $p < 0.05$; Student's *t*-test).

gFW, gram fresh weight; tZ, *trans*-zeatin; tZR ,tZ riboside; tZRPs, tZ ribotides; cZ, cis-zeatin; cZR, cZ riboside; cZRPs, cZ ribotides; DZ, dihydrozeatin; DZR, DZ riboside; DZRPs, DZ ribotide; iP, N6-(Δ2-isopentenyl)adenine; iPR, iP riboside; iPRPs, iP ribotides; tZ7G, tZ-7-N-glucoside; tZ9G, tZ-9-N-glucoside; tZOG, tZ-O-glucoside; cZOG, cZ-O-glucoside; tZROG, tZR-O-glucoside; cZROG, cZR-O-glucoside; DZ9G, DZ-9-N-glucoside; iP7G, iP-7-N-glucoside; iP9G, iP-9-N-glucoside; iP-type CK precursors, sum of iP, iPR and iPRPs; tZ-type CK precursors, sum of tZ, tZR and tZRPs; N.D., not detected.

Supplementary Table S8. Cytokinin concentration in shoots and roots of Col-0, *ipt3 ip5 ipt7* and *cyp735a1 cyp735a2* seedlings exposed to low or high CO_2

Shoots and roots were harvested from 12 day-old seedlings grown on 1/2MS agar plates at 280 ppmv and then exposed to 280 ppmv or 780 ppmv CO_2 for 24h. Data are means ± standard deviation (n = 3).

pmol/gFW	Col-0				*ipt3 ipt5 ipt7*				*cyp735a1 cyp735a2*			
	Shoot		Root		Shoot		Root		Shoot		Root	
	280 ppmv	780 ppmv	280 ppmv	780 ppmv	280 ppmv	780 ppmv	280 ppmv	780 ppmv	280 ppmv	780 ppmv	280 ppmv	780 ppmv
tZ	0.46±0.01	0.65±0.06**	1.85±0.14	2.92±0.27**	0.1±0.01	0.11±0.01	0.37±0	0±0	N.D.	N.D.	N.D.	N.D.
tZR	0.23±0.03	0.54±0.04**	3.06±0.17	6.59±1.09**	N.D.	N.D.	0.33±0.01	0.33±0.06	N.D.	N.D.	0.07±0.03	0.12±0.09
tZRPs	2.61±0.62	5.03±0.32**	5.09±0.46	9.55±0.78**	0.38±0.03	0.5±0.05**	0.7±0.13	0.67±0.17	0.22±0.05	0.43±0.16	0.19±0.13	0.33±0.12
cZ	0.15±0.04	N.D.	0.7±0.11	N.D.	0.13±0.05	N.D.	1.16±0.13	0.62±0.1	0.16±0.03	N.D.	0.77±0.16	0.71±0.2
cZR	0.29±0.08	0.1±0.01*	2.3±0.47	1.94±0.75	0.17±0.04	0.09±0*	7.61±0.71	5.87±1.82	0.25±0.04	0.13±0.01*	4.87±3.63	4.19±1.27
cZRPs	1.7±0.29	0.84±0.07*	6.51±1.23	5.72±1.79	1.09±0.1	0.68±0.06	16.37±1.6	13.76±1.8	1.63±0.25	1.02±0.07*	11.45±2.88	10.53±0.9
DZ	N.D.	N.D.	N.D.	N.D.	N.D.	N.D.	N.D.	N.D.	N.D.	N.D.	N.D.	N.D.
DZR	N.D.	N.D.	N.D.	N.D.	N.D.	N.D.	N.D.	N.D.	N.D.	N.D.	N.D.	N.D.
DZRPs	N.D.	N.D.	N.D.	N.D.	N.D.	N.D.	N.D.	N.D.	N.D.	N.D.	N.D.	N.D.
iP	0.06±0	0.06±0	0.18±0.01	0.19±0.01	0.03±0	0.03±0	0.12±0	0.14±0.02	0.1±0.01	0.11±0.01	0.67±0.06	0.72±0.01
iPR	0.02±0	0.02±0	0.13±0.02	0.19±0.02*	0.01±0	0.01±0	0.04±0.01	0.05±0.01	0.04±0.01	0.08±0.01*	0.7±0.49	1.25±0.19
iPRPs	13.66±1.53	16.46±0.5*	6.27±0.04	8.2±0.75*	5.67±0.14	5.22±0.54	2.68±0.23	3.76±0.3**	20.72±1.2	25.18±1.54	30.08±1.96	39.42±1.95
tZ7G	17.01±0.68	15.56±0.75*	27.64±1.82	32.15±1.97*	2.36±0.21	2.26±0.19	3.08±0.18	3.02±0.22	0.89±0.04	1.16±0.41	0.79±0.09	1.2±0.89
tZ9G	8.54±0.44	7.77±0.17*	19.02±0.98	22.47±0.97*	1.06±0.09	1.03±0.1	2.21±0.19	2.17±0.12	0.3±0.02	0.48±0.21	0.31±0.08	0.65±0.64
tZOG	5.03±0.15	5.7±0.36*	6.17±0.21	7.97±0.76*	1.15±0.03	1.16±0.09	1.53±0.08	1.55±0.07	0.44±0.03	0.74±0.41	N.D.	N.D.
cZOG	N.D.	N.D.	N.D.	N.D.	1.13±0.06	1.11±0.05	2.44±0.03	2.44±0.08	1.39±0.06	1.22±0.08	3.32±0.24	3.05±0.18
tZROG	0.14±0.01	0.14±0.02	0.08±0.01	0.1±0.02	0.03±0	0.03±0	0.04±0.01	0.04±0.01	0.02±0	0.02±0	0±0	0±0
cZROG	1.73±0.13	1.58±0.15	0.18±0.01	0.17±0.02	1.46±0.12	1.36±0.07	0.23±0.03	0.21±0	1.65±0.06	1.44±0.06	0.24±0.03	0.21±0.02
tZRPsOG	N.D.	N.D.	N.D.	N.D.	N.D.	N.D.	N.D.	N.D.	N.D.	N.D.	N.D.	N.D.
cZRPsOG	N.D.	N.D.	N.D.	N.D.	N.D.	N.D.	N.D.	N.D.	N.D.	N.D.	N.D.	N.D.
DZ9G	0.13±0	0.12±0.01	0.18±0.01	0.2±0.02	0.05±0	0.05±0	N.D.	N.D.	N.D.	N.D.	N.D.	N.D.
iP7G	17.03±0.88	17.04±0.54	7.58±0.25	8.2±0.28*	5.81±0.24	5.51±0.12	3.71±0.13	3.65±0.03	24.53±0.36	22.9±0.59	37.04±3.03	35.28±2.55
iP9G	3.72±0.19	3.74±0.08	1.38±0.06	1.57±0.07*	1.23±0.06	1.17±0.05	0.56±0.01	0.56±0.03	5.82±0.08	5.41±0.21	10.96±1.01	10.06±0.6
iP-type CK precursors	13.68±1.53	16.48±0.5*	6.4±0.06	8.39±0.77*	5.68±0.14	5.23±0.54	2.72±0.24	3.81±0.31	20.76±1.21	25.26±1.55**	30.78±2.45	40.67±2.14**
tZ-type CK precursors	2.84±0.65	5.57±0.36**	8.15±0.63	16.14±1.87**	0.38±0.03	0.5±0.05*	1.03±0.14	1±0.23	0.22±0.05	0.43±0.16	0.26±0.16	0.45±0.21

, statistically significant differences between 280 ppmv CO_2- and 780 ppmv CO_2-treated samples of the same genotype (, $p < 0.05$; **, $p < 0.01$; Student's *t*-test).

gFW, gram fresh weight; tZ, *trans*-zeatin; tZR ,tZ riboside; tZRPs, tZ ribotides; cZ, cis-zeatin; cZR, cZ riboside; cZRPs, cZ ribotides; DZ, dihydrozeatin; DZR, DZ riboside; DZRPs, DZ ribotide; iP, N6-(Δ2-isopentenyl)adenine; iPR, iP riboside; iPRPs, iP ribotides; tZ7G, tZ-7-N-glucoside; tZ9G, tZ-9-N-glucoside; tZOG, tZ-O-glucoside; cZOG, cZ-O-glucoside; tZROG, tZR-O-glucoside; cZROG, cZR-O-glucoside; DZ9G, DZ-9-N-glucoside; iP7G, iP-7-N-glucoside; iP9G, iP-9-N-glucoside; iP-type CK precursors, sum of iP, iPR and iPRPs; tZ-type CK precursors, sum of tZ, tZR and tZRPs; N.D., not detected.

Supplementary Table S9. List of primers used for quantitative PCR analysis

Gene name	Locus ID	Forward (5' to 3')	Reverse(5' to 3')
CYP735A1	At5g38450	GGCCATGGTTTCGCAATC	CCGTTTCCGTTAAGCAAAGC
CYP735A2	At1g67110	ATGGTGTCCCTTCCGTTGAACA	GAGGGTAAAGTCTTAATGACTCGT
AtIPT1	At1g68460	AGAGATCACAACGAATCAGATTACGT	ATGACGCCGAGGAGATGGT
AtIPT3	At3g63110	CATGGCGAATCTCTCCATTGA	AGTTGGAACCTCCAACGATGA
AtIPT4	At4g24650	CACCACGAATCAATCGACCAT	GAAATTCTGCCGCTGTGACTT
AtIPT5	At5g19040	AGGATTTTCAGCGTGAAGCAA	CTATGATCGGGACACGGTCTCT
AtIPT6	At1g25410	GCAATCGTGTCGACCAAATG	CCTAGTCGTCGACCCGAATC
AtIPT7	At3g23630	AACCTAACGGCCACCCAGTA	TGTTGTTCGCTGAGAGTTTCGA
AtIPT8	At3g19160	TTCCCATGACTACCGTTTGCA	CGATTGAGAGGCATGACTTGC
CKX1	At2g41510	GCTTGGACAGTTTGGCATAAT	TGGTCCCTTGAAAATGCAGA
CKX3	At5g56970	ACAGTCGGTGGGACGTTATC	CCTAACACCGCGAAGAAAAG
CKX4	At4g29740	GTTTAGACACGGCCCTCAGA	CAATCCTGGCCCTCGTTAT
CKX5	At1g75450	AGATCACCAAGAACTACCACG	GTCGCTGTTCTGCCTCTTTG
CKX6	At3g63440	ATTCCGACATGGACCACAGA	TCCGTTGTAAAGACCGATGTC
CKX7	At5g21482	TGGTATCATCACCAGAGCTAGG	ACTCGGCGTCTTGAGTGAACTC
ARR4	At1g10470	GTTGACTGTTTCGACTGAATC	GAAGTTATGCTACCGAGGAAG
ARR6	At5g62920	GAAGTTATGCTACCGAGGAAG	TACGATCAACGTGACTGTCGT
ARR15	At1g74890	ATCTCCATCATCATCATCAAC	GACTCTAATTTGATCCTCTTGG
ABCG14	At1g31770	ATCTGTTACTACACTCGTTTTTCTC	TGTAGCAGTAGTAGCTGTAGCTTAG
At4g34270	At4g34270	GTGAAAACTGTTGGAGAGAAGCAA	TCAACTGGATACCCTTTCGCA
